# Alpha-7 nicotinic acetylcholine receptor: targeting the interplay between inflammation, renin-angiotensin aldosterone system, and nervous system for the novel treatment of heart failure

**DOI:** 10.3389/fphar.2026.1756039

**Published:** 2026-03-19

**Authors:** Jordan Swiderski, Laura Kate Gadanec, Stephen Hearth, Benjamin Darcy Rowlands, Andrew James Murphy, Vasso Apostolopoulos, Anthony Zulli

**Affiliations:** 1 Institute for Health and Sport (IHES), Victoria University, Melbourne, VIC, Australia; 2 School of Health Sciences, The University of Notre Dame Australia, Sydney, NSW, Australia; 3 Division of Immunometabolism, Baker Heart and Diabetes institute, Melbourne, VIC, Australia; 4 School of Health and Biomedical Science, RMIT University, Melbourne, VIC, Australia

**Keywords:** cardiovascular disease, cholinergic anti-inflammatory pathway, heart failure, hypertension, renin-angiotensin-aldosterone system, α7 nicotinic acetylcholine receptor

## Abstract

The incidence and prevalence of heart failure (HF) with preserved ejection fraction (HFpEF) continue to rise, yet evidence-based therapy remains limited. Due to the complexity of HFpEF pathology, traditional HF medication has shown inconsistent efficacy in improving clinical outcomes and reducing morbidity. Therefore, highlighting the urgent need for novel interventions. The αlpha-7 nicotinic acetylcholine receptor (α7nAChR) is a central mediator of the cholinergic anti-inflammatory pathway and has emerged as a promising therapeutic target in various conditions, such as sepsis, arthritis, metabolic dysfunction, and atherosclerosis. This review aims to examine the emerging therapeutic potential of α7nAChR in HF and HFpEF pathology, focusing on its protective role in modulating the complex interplay between systemic and cardiovascular inflammation, renin-angiotensin-aldosterone system activation, neurocardiac signaling and metabolic dysfunction.

## 1 Introduction

**FIGURE 1:**
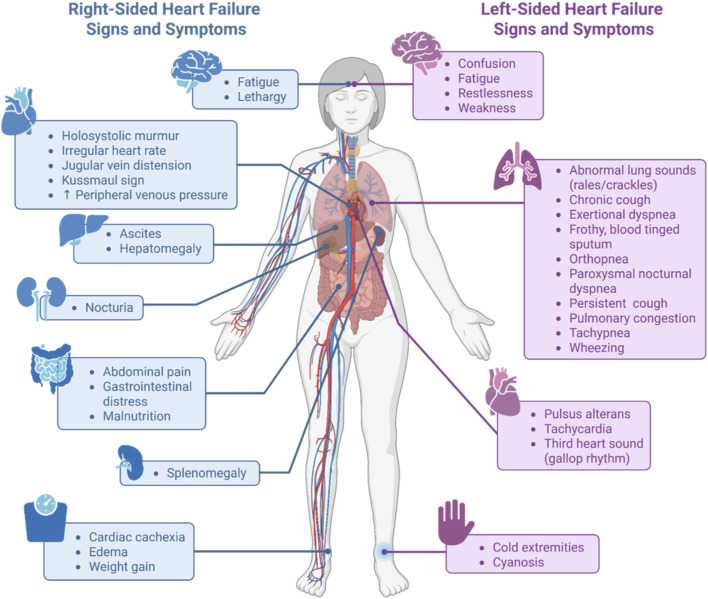
Conceptual schematic of common signs and symptoms of left- and right-sided HF. HF arises from structural and/or functional abnormalities of the heart, resulting in elevated intracardiac pressures, weakening of the heart muscle, and/or inadequate cardiac output that occur at rest and/or during physical activity (McDonagh et al., 2021). Non-invasive physical examination remains important in managing HF, and patients presenting with left- or right-sided HF display distinct signs and symptoms that may aid in the diagnosis and treatment strategy (Dini et al., 2023; Thibodeau and Drazner, 2018). Image created with BioRender.com.

Cardiovascular diseases (CVDs) comprise a broad range of disorders affecting the heart and blood vessels, including atherosclerosis, coronary artery disease, myocardial infarction, hypertension, and heart failure (HF) (Roth et al., 2020). Among these, HF is a leading cause of morbidity and mortality, affecting over 64 million people worldwide (Norhammar et al., 2023; Savarese et al., 2022). In western populations, HF prevalence rises from approximately 1% in individuals under 55 years to 10% in those over 70 years (McDonagh et al., 2021). The European Society of Cardiology categorizes HF based on left ventricle ejection fraction (LVEF) into three groups: HF with preserved (HFpEF; LVEF ≥50%), reduced (HFrEF; LVEF <40%), and mildly reduced ejection fraction (HFmrEF; LVEF 40%–49%) (McDonagh et al., 2021). These guidelines are used to assist healthcare professionals in diagnosing, managing symptoms (Figure 1), and selecting treatment strategies for patients suffering from acute and chronic HF.

**FIGURE 2:**
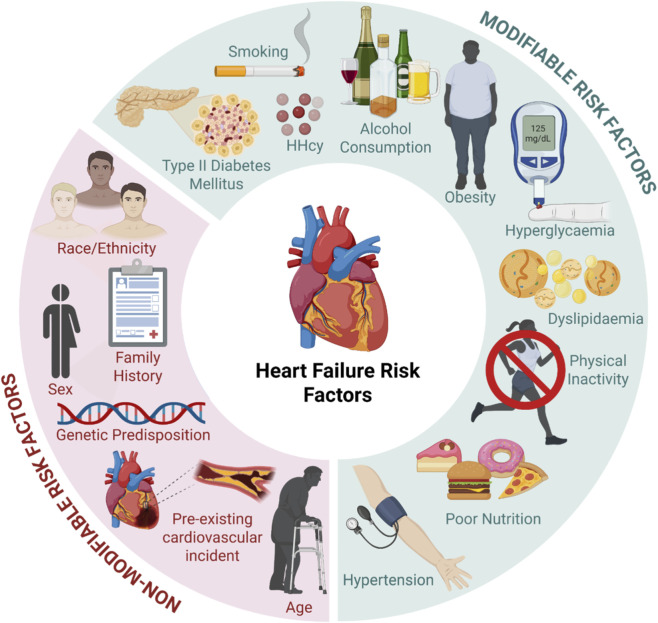
Non-modifiable and modifiable risk factors correlated with HF. Non-modifiable risk factors are characteristics that are unable to be altered and influence an individual’s likelihood of developing HF, and include age (Tromp et al., 2021), ethnicity (Savitz et al., 2021), family history (Rasooly et al., 2023), genetic predisposition (Lee DS. et al., 2025), pre-existing cardiovascular incident (e.g., atherosclerosis, coronary artery disease, myocardial infarction, and peripheral artery disease) (Brar et al., 2025; Samsky et al., 2021) and sex (Lala et al., 2022). Conversely, modifiable risk factors are behaviors and traits that can be changed and controlled, and impact an individual’s probability of developing HF, including alcohol consumption (Lee D-I. et al., 2025), dyslipidemia (Nebuwa et al., 2024), hyperglycemia (Chioncel and Ambrosy, 2020), hyperhomocysteinemia (HHcy) (Karger et al., 2025), hypertension (Baffour et al., 2024), obesity (Rayner et al., 2025), poor diet (Billingsley et al., 2020) physical inactivity (Aune et al., 2021), smoking (Watson et al., 2019) and type II diabetes mellitus (Kenny and Abel, 2019). Image created with BioRender.com.

HFpEF is characterized by impaired diastolic relaxation and elevated pressure despite preserving systolic function (Naing et al., 2019). HFpEF accounts for approximately 50% of HF cases, with its prevalence rising significantly over the past few decades (Vasan et al., 2018). Currently, there is a lack of evidence-based interventions for HFpEF, partly due to its heterogeneous nature, which makes the condition difficult to target and effectively manage. Underlying aetiologias are driven by various modifiable and non-modifiable factors, as summarized in Figure 2, and include age (Tromp et al., 2021), sex (Lala et al., 2022), genetics (Lee DS. et al., 2025), obesity (Rayner et al., 2025), diabetes, and pre-existing cardiovascular conditions (Brar et al., 2025; Bahit et al., 2018; Samsky et al., 2021). While many pre-existing cardiovascular conditions are associated with HF, hypertension remains the highest risk factor for HFpEF, particularly in the elderly. Despite therapeutic advances, the overall 5-year survival rate of HF is approximately 50% (Zhou et al., 2024; Khan et al., 2024). Thus, it stands to reason that treatments targeting the underlying risk factors and conditions may help stem the growing global burden of HF. While the use of renin-angiotensin-aldosterone system (RAAS) inhibitors, beta blockers, and mineralocorticoid (aldosterone) receptor antagonists are mainstays in the treatment of HF (McDonagh et al., 2021), these anti-hypertensive agents have failed to show consistent prognostic improvements in HFpEF (Rist et al., 2024; Beldhuis et al., 2017; Odajima et al., 2022; Conraads et al., 2012; Pitt et al., 2014; Solomon et al., 2019; Yamamo et al., 2013). Therefore, there is undoubtedly an urgent need for additional effective therapeutic strategies.

The alpha-7 nicotinic acetylcholine (ACh) receptor (α7nAChR) is a key protein of the cholinergic anti-inflammatory pathway, which links the nervous and inflammatory systems (Noviello et al., 2021). This pathway has gained significant attention for its therapeutic potential in several inflammatory conditions, including atherosclerosis (Vieira-Alves et al., 2020), cancer (Arunrungvichian et al., 2023), neurodegenerative disorders (Let et al., 2022; Yang et al., 2017), and rheumatoid arthritis (Wu et al., 2025). HF with HFpEF is increasingly recognized as the result of a complex interplay between low-grade systemic inflammation, RAAS activation, microvascular injury, metabolic dysregulation, and neural dysfunction (Hartupee and Mann, 2017). These processes promote myocardial damage through pathological cardiac remodeling (i.e., ventricular wall thickening and fibrosis), inflammation, oxidative stress, and increased myocyte apoptosis (Hartupee and Mann, 2017).

Given that interconnected pathways are increasingly explored for novel treatments (Bauersachs et al., 2025), this review seeks to examine the emerging role of α7nAChR in the interplay between anti-inflammatory signaling, the RAAS, and neurocardiac and metabolic regulation to determine its therapeutic potential for HF and HFpEF. To our knowledge, this is the first review to consolidate experimental and clinical evidence, directly linking α7nAChR signaling to HF pathology, with an emphasis on HFpEF. Relevant peer-reviewed publications focusing on α7nAChR signaling in CVDs, HF, and associated pathology were used.

## 2 Physiology of α7nAChR

The ACh receptor (AChR) is a well-characterized cholinergic membrane receptor that mediates the physiological responses of ACh across neuronal and non-neuronal pathways (Reichrath et al., 2016; Saw et al., 2021; Wessler and Kirkpatrick, 2008). AChRs are broadly classified into two types: (i) muscarinic AChRs, which are primarily involved in parasympathetic nervous system signaling, and (ii) nicotinic AChR (nAChR), a family of ligand-gated ion channels comprising 17 different subunits (ɑ1-ɑ10, β1-β4, *γ*, *δ*, and ε), each contributing to diverse physiological roles (Xu et al., 2021). Among these, the α7nAChR, encoded by the CHRNA7 gene, is one of the most abundantly expressed subtypes (Xu et al., 2021). Initially identified in the central nervous system, ɑ7nAChRs were observed to play an important role in mediating fast synaptic transmissions, neurotransmitter release, and ion influx to regulate cognitive processes (Barrantes et al., 1994; Rathouz and Berg, 1994). Compared to other nAChR subtypes, ɑ7nAChR displays unique characteristics, including high Ca^2+^ permeability and rapid activation-desensitization kinetics (Xu et al., 2021). The receptor exists in three functional states: resting (closed), active (open), and desensitized (closed) (Noviello et al., 2021). Beyond its role in neurons, more recently, ɑ7nAChR expression has also been documented on a variety of non-neuronal cells, including hepatocytes (Li et al., 2019), cardiomyocytes (Vang et al., 2021; Broide et al., 2019), cardiac fibroblasts (Li et al., 2019), endothelial cells (Li et al., 2019; Broide et al., 2019), vascular smooth cells (Li et al., 2019; Wada et al., 2007), stromal cells and immune cells (e.g., leukocytes, macrophages, dendritic cells, T-cells, and B-cells) (Schloss et al., 2022; Keever et al., 2024; Mashimo et al., 2019), where it plays a critical role in physiological homeostasis.

### 2.1 Activation and inhibition of α7nAChR

Despite its therapeutic potential, direct activation of ɑ7nAChR using endogenous agonists, such as ACh and nicotine, remains challenging. Nicotine is a non-selective agonist of nAChR subtypes, with known toxicity and addictive properties that restrict its clinical use (Cao et al., 2024). Meanwhile, ACh, is rapidly degraded by cholinesterases, such as acetylcholinesterase (AChE) and butyrylcholinesterase (BChE) and lacks receptor specificity (Liu et al., 2024). Early pharmacological efforts focused on orthostatic ligands, compounds that bind to the orthostatic site within the receptor and induce partial or full agonism, mimicking ACh by inducing ion channel opening, as well as competitive antagonists, which block receptor activity (Yang et al., 2017; Manetti et al., 2023) (Table 1). More recently, a significant advancement in ɑ7nAChR pharmacology is the development of positive allosteric modulators (PAMs). Unlike orthostatic agonists, PAMs bind to distinct allosteric sites on the extracellular domain and enhance the receptor responsiveness to ligands without directly activating the channel (Hurst et al., 2005; Papke and Horenstein, 2021). PAMs, such as PNU-120596, cannot induce receptor agonism on their own; instead they reduce the refractory period of ɑ7nAChR desensitization to enhance receptor responsiveness in the presence of endogenous or orthostatic agonists (Mueller et al., 2015; Jin et al., 2014). In contrast, PAMs like GAT-107 can activate ɑ7nAChR independently by acting on both the orthostatic and allosteric sites, offering dual-action potential (Bagdas et al., 2016; Gauthier et al., 2021). Although ɑ7nAChR antagonists have limited therapeutic value, they have been instrumental in identifying the biological role of ɑ7nAChR. ɑ-Bungarotoxin (ɑ-BTX), is a neurotoxin derived from the venom of the Many-banded Krait snake (Bungarus multicinctus), which irreversibly blocks ɑ7nAChR, while methyllycaconitine (MLA) is a widely used competitive antagonist known for its specificity in experimental models (Panagis et al., 2000; Prickaerts et al., 2012).

**TABLE 1:** Cholinergic agonists and antagonists that target α7nAChR.

Compound	Classification	Chemical structure	Clinical status
ɑ7nAChR agonists			
AR-R17779	Selective, full agonist (Fan et al., 2014); may cross react with 5-HT_3_ receptor (Hammarlund et al., 2021).	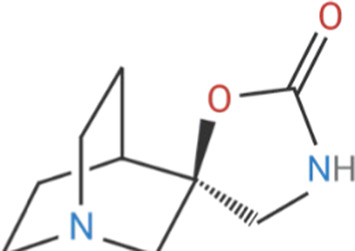	N/A
AZ6983	Highly specific; selective over 5-HT_3a_R and α3β4nAChR (Ulleryd et al., 2019).	Chemical structure undisclosed.	N/A
GAT-107	Strong dual agonist and PAM (Bagdas et al., 2016; Papke et al., 2014).	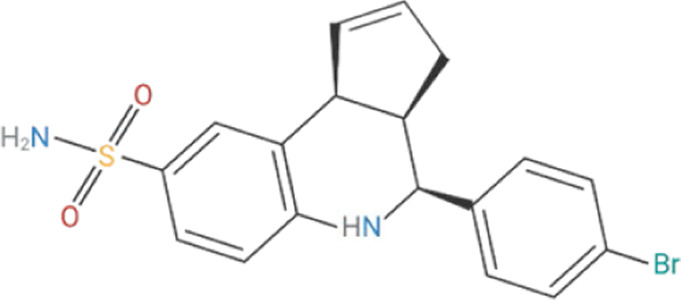	N/A
GTS-21	Partial and long-lasting agonist; weak α4β2 and 5-HT_3_ antagonist; also activates α3β4 nAChRs (Yang et al., 2017; Papke and Horenstein, 2021).	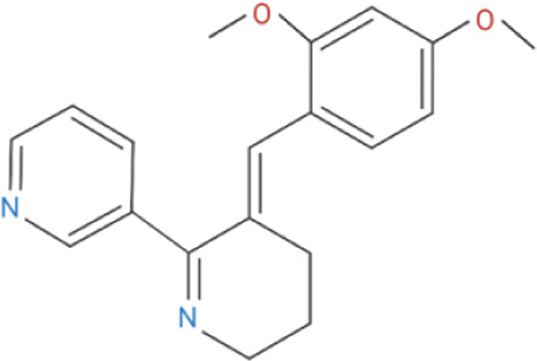	Phase I (Obesity) NTC02458313 (2016) Phase I (Inflammation) NCT00783068, 2010 Phase II (Cognition) NCT00414622, 2007
Nicotine	Non-selective agonist; desensitizes with prolonged exposure (Yang et al., 2017; Wittenberg et al., 2020).	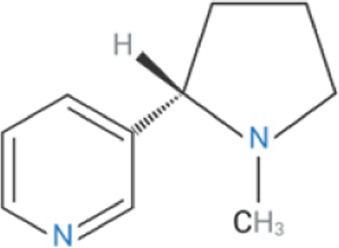	​
PHA-543613	Potent and selective agonist; may promote desensitization (Wishka et al., 2006; B et al., 2019).	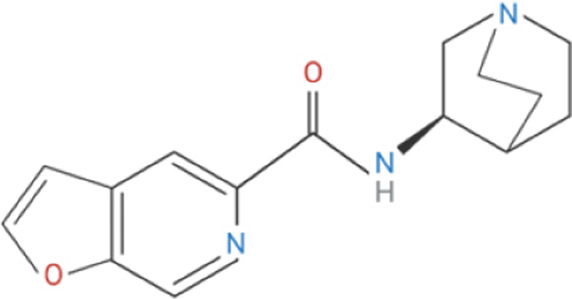	N/A
PNU-120596	Potent and selective PAM; prevents nicotine-induced desensitization (Kalappa and Uteshev, 2013; Hurst et al., 2005).	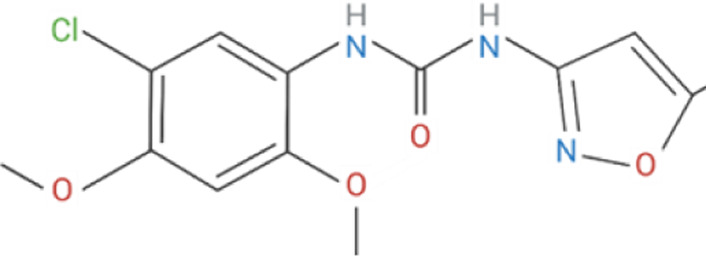	N/A
PNU-282987	Potent; selective over α3β4nAChR; rapid desensitization (Bodnar et al., 2005); functional 5-HT_3_ antagonist (Bodnar et al., 2005).	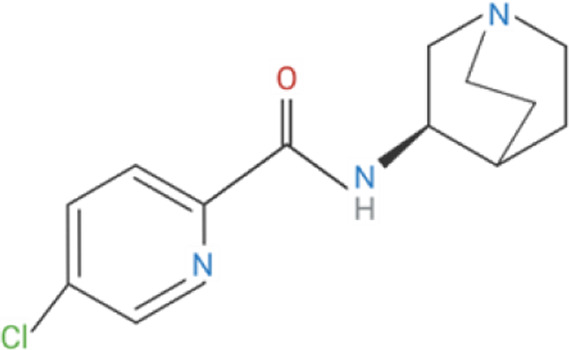	N/A
BTXɑ7nAChR antagonists			
ɑ-BTX	Non-selective for ɑ7 and muscular nAChRs (Noviello et al., 2021); reported as non-competitive; locks binding site inactive (Dacosta et al., 2015).	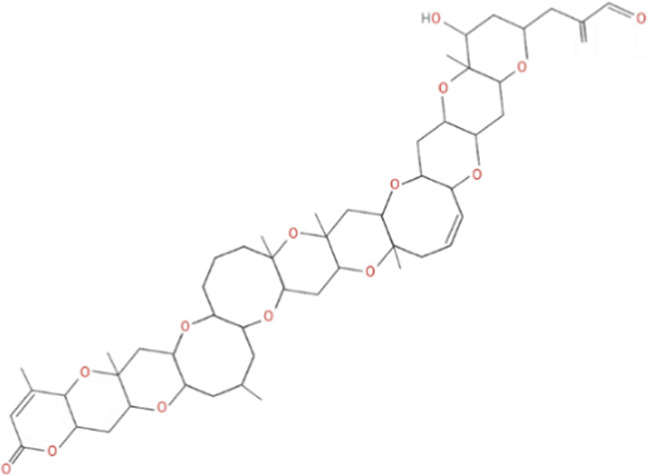	​
MLA	Highly potent and selective competitive antagonist (Qasem et al., 2023).	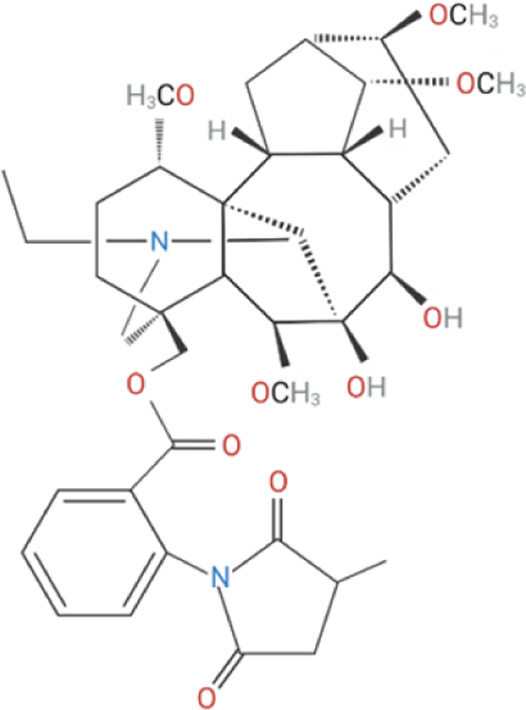	​

Abbreviations: α7nAChR, alpha 7 nicotinic acetylcholine receptor; Br, bromide; C, carbon; Cl, chloride; H, hydrogen; N, nitrogen; nAchR, nicotinic acetylcholine receptor; O, oxygen; PAM, positive allosteric modulator; 5-HT_3_R, serotonin type-3, receptors.

## 3 α7nAChR and inflammation

Overwhelming evidence supports that acute and chronic low-grade inflammation plays a central role in the pathogenesis, progression, and severity of HF (Boulet et al., 2024). Systemic and local inflammation have a detrimental effect on myocardial structure and function, with inflammatory mediators enhancing oxidative damage to myocardial cells, fibrosis, ventricular stiffness, and coronary microvascular dysfunction (Bairey Merz et al., 2020; Paulus and Tschope, 2013). Clinical studies report that HFpEF severity is correlated with an elevation in both circulating and cardiac pro-inflammatory cytokines and immune modulators (interleukin (IL)-1β, IL-6, IL-17, tumour necrosis factor (TNF), monocyte chemoattractant protein-1 (MCP-1), nitric oxide (NO), inducible nitric oxide synthase (iNOS), high-mobility group box-1 (HMGB-1) and C-reactive protein) (Chirinos et al., 2020; Delalat et al., 2025; DuBrock et al., 2018; Sanders-van Wijk et al., 2020); infiltration of immune cells [e.g., monocytes, macrophages, and lymphocytes (T and B)] (Kessler et al., 2025; Kneuer et al., 2025; Li et al., 2010); and expression of inflammatory receptors [e.g., pattern recognition receptors (PRR), such as toll-like receptors (TLRs) and receptor for advanced glycation end-products (RAGE)] (Delalat et al., 2025; Daou et al., 2023). Considering the underlying involvement of inflammation in the pathophysiology of HF, targeting aspects of the immune system has been seen as a viable approach to improving disease severity and patient outcome (Mesquita et al., 2021; Ma et al., 2022; Bashier et al., 2019; Petrie et al., 2018).

**FIGURE 3:**
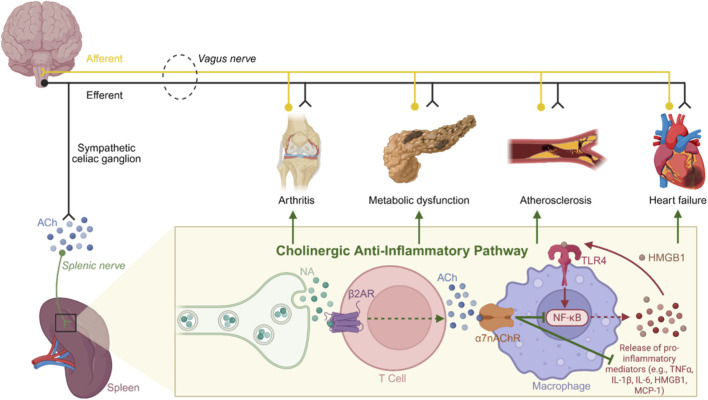
Schematic diagram of the proposed cholinergic anti-inflammatory pathway. The cholinergic anti-inflammatory pathway stems from activation of efferent vagus nerve (black). This results in the release of ACh within the celiac ganglion to activate sympathetic splenic nerves, triggering the release of noradrenaline (Xie et al., 2020). Noradrenaline then binds to beta-2 adrenergic receptors on T cells resulting in the release of ACh (Xie et al., 2020). ACh activates a7nAChRs located on macrophages and downregulates the release of pro-inflammatory cytokines and immune mediators by inhibiting nuclear activity of NF-κB by immune receptors, such as TLR4 (110) (Simon et al., 2023). Thus, targeting the cholinergic anti-inflammatory pathway is an attractive therapeutic option for the treatment of pathologies, including arthritis (Wu et al., 2025), metabolic dysfunction (Wu YJ. et al., 2021), atherosclerosis (Vieira-Alves et al., 2020) and HF, where chronic inflammation is a hallmark of the disease. Abbreviatiosn: αlpha-7 nicotinic acetylcholine receptor, α7nAChR; ACh, acetylcholine; IL, interleukin; β2AR, beta-2 adrenergic receptor; HMGB1, high-mobility group box-1; NA, noradrenaline; NF-κB, Nuclear factor kappa-light-chain-enhancer of activated B-cell; TNF-α, tumor necrosis factor alpha. Image created with BioRender.com.

Interest in the ɑ7nAChR as a target for suppressing adverse inflammatory responses was first identified in 2003 when it was established that stimulation of the vagus nerve attenuated lipopolysaccharide (LPS)-induced inflammation by inhibiting TNF-ɑ release (Borovikova et al., 2000). Subsequently, a series of α7nACh knockout models confirmed that neuronal and non-neuronal ɑ7nAChR signaling was essential to this anti-inflammatory response and was termed the “cholinergic anti-inflammatory pathway” (Figure 3) (Keever et al., 2024; Wang et al., 2003; Huston et al., 2006; Hoogland et al., 2022; Truong et al., 2015). Originating in the brainstem as a response to afferent vagus nerve stimulation by inflammation, the cholinergic anti-inflammatory pathway consists of neurotransmitter release from the efferent arm of the vagus nerve to the splenic nerve, resulting in noradrenaline (NA) release from splenic cells (Keever et al., 2023). NA binds to adrenergic receptors on the surface of splenic T-cells and B-cells to increase the production of choline acetyltransferase (ChAT), an enzyme responsible for the production and subsequent release of ACh (Keever et al., 2023). ACh then binds to and activates surface ɑ7nAChR on themselves or on macrophages to reduce inflammation by suppressing the release of inflammatory cytokines (i.e., TNF-ɑ, IL-1β, and IL-6), chemokines (i.e., intracellular adhesion molecule-1, vascular cell adhesion-1 and molecule, and MCP-1), and danger associated molecular pattern molecules (DAMPs), such as high-mobility group box-1 (HMGB1) (Mashimo et al., 2019; Keever et al., 2023; Rosas-Ballina et al., 2011; Vieira-Alves et al., 2020; Reardon et al., 2013; Fujii et al., 2017; Zhang W. et al., 2022). Therefore, selective activation of α7nAChR may provide effective inhibition of damaging inflammatory signaling in critical conditions (Table 2).

**TABLE 2:** Role of α7nAChR in mitigating inflammation.

Model	Intervention	Outcome	References
*In vivo* encephalomyelitis-induced inflammation in female C57BL/6 mice	Treated with nicotine 2 mg/kg/d, 28 days	↓ Circulation of TNF-α and IFN-γ ↓ NF-κB transcription ↓ T-cell proliferation	Nizri et al. (2009)
*In vitro* LPS-induced inflammation in RAW264.7 macrophages	Incubation of GTS-21 1–10µM, 16 h	↓ Expression of TNF and HMGB1	Yang et al. (2019)
*In vitro* LPS-induced inflammation in isolated human peripheral monocytes and U937 monocytes	Incubation of nicotine 10µM, 1 h	↓ Expression of TNF-α, PGE_2_, and COX-2 ↓ IκBα phosphorylation ↓ NF-κB transcriptional activity	Yoshikawa et al. (2006)
*In vivo* encephalomyelitis induced in female C57Bl/6 mice	Treated with GAT-107 10 mg/kg/d, 9 days	↓ Circulation of IL-6, IL-17, and IFN-γ ↑ IL-10 ↓ Lymphocyte proliferation ↓ B-cells	Mizrachi et al. (2021)
*In vivo* TNBS-induced colitis in female CD1 Swiss mice	Treated with AR-17779 1.5 mg/kg	↓ Expression of IL-1β and IL-6 ↓ T-cells ↓ Macrophages	Grandi et al. (2017)
*In vivo* hydroxydopamine induced inflammation in male Wister rats	Treated with PNU-282987 3 mg/kg	↓ Expression of TNF-α and IL-1β ↑ α7nAChR expression	Jiang et al. (2020)

Abbreviations: α7nAChR, alpha 7 nicotinic acetylcholine receptor; ATP, adenosine triphosphate; HMGB1, high-mobility group box-1; IFN-γ, interferon gamma; IL, interleukin; LPS, lipopolysaccharide; mtDNA; mitochondrial DNA; NF-κB, nuclear factor kappa beta; PGE_2_, prostaglandin E2; TNF-α, tumour necrosis factor alpha, ↑, increase; ↓ decrease.

### 3.1 Immune cells

Increased circulation and infiltration of immune cells (macrophages and lymphocytes) is a hallmark characteristic in the early phases of HFpEF (Bhagat et al., 2022; Strassheim et al., 2019). In brief, vascular endothelial and myocardial cell damage can favor the recruitment and infiltration of monocytes and macrophages into tissue, leading to the activation and amplification of local inflammatory mechanisms that can cause fibrotic remodeling and cellular death (Glezeva and Baugh, 2014; Hulsmans et al., 2018). Moreover, an influx of T-cells in damaged cardiac tissue has been implicated in chronic HFpEF development through the generation of autoimmune responses that facilitate vicious and unregulated cycles of inflammatory damage and fibrosis (Aghajanian et al., 2019; Blanton et al., 2019; Kalli et al., 2017).

It is well established that ɑ7nAChR is highly expressed on the surface of macrophages and T-cells, where it contributes to the regulation of inflammatory processes (Wang et al., 2003; Sato et al., 1999). Various publications have established that ACh, nicotine, and ɑ7nAChR agonists, such as GTS-21, inhibit the release of inflammatory cytokines (i.e., IL-1β, IL-6, IL-17, and TNF) from macrophages (Mizrachi et al., 2021; Abot et al., 2018; Garg and Loring, 2019). In the context of atherosclerosis, administration of GTS-21 was shown to reduced plaque size, which was associated with a decrease in circulating monocytes through reduced splenic extramedullary myelopoiesis and inflammation (Schloss et al., 2022; Al-Sharea et al., 2017). Moreover, it appears that activation of ɑ7nAChR in bone marrow stromal cells is required for normal hematopoiesis, and its deletion enhances myeloid cell production. Thus, activating the ɑ7nAChR produces effects that are therapeutically desirable for the treatment of HFpEF.

Indeed, the therapeutic potential of ɑ7nAChR restoring cardiac dysfunction has been shown through allosteric modulator PNU-282987, which protected mice from stroke-induced HF by modulating proliferation of macrophages away from an inflammatory phenotype and reducing subsequent cardiac expression of inflammatory mediators, including IL-1β, MCP-1, TNF, TLR4, and HMGB1 (Su et al., 2022). These effects were completely reversed by co-administration of receptor antagonist MLA (Su et al., 2022). T-cell accumulation within the heart is also a characteristic of both human and animal HFpEF, and is associated with the release of TNF and IFN-γ, which can upregulate signaling pathways to promote cardiac hypertrophy, fibrosis, and dysfunction (Huang et al., 2021; Kumar et al., 2022; Westermann et al., 2011). Inhibiting T-cell activation and infiltration has been suggested to be a viable therapy in the prevention of cardiomyocyte death in mice with HF (Kalli et al., 2017). The regulatory role of ɑ7nAChR in T-cell activation is supported by several studies demonstrating that receptor inhibition is associated with the activation and enhanced proliferation of pro-inflammatory T-cells (Nizri et al., 2006; Nizri et al., 2008; Zdanowski et al., 2015). Moreover, activation of ɑ7nAChR by GTS-21 has been shown to suppress isolated T-cell activation and reduces IFN-γ production (Wu et al., 2014), which may be therapeutically relevant in the mitigation cardiac fibrosis (Nevers et al., 2017). These studies demonstrate the immunosuppressive role of ɑ7nAChRs in modulating immune function through regulation of immune cells and cytokine production and may be relevant for targeting the hyper-inflammatory responses characterized by CVDs and HF.

### 3.2 Anti-inflammatory pathways

Nuclear factor kappa-light-chain-enhancer of activated B-cell (NF-κB) is a key transcription regulator that drives inflammatory, fibrotic, and apoptotic genes (Lee et al., 2003). Its activation can be triggered by RAAS, reactive oxygenated species, inflammatory cytokines, DAMPs, and TLR4 ligands (Liu T. et al., 2017; Chauhan et al., 2022; Cantero-Navarro et al., 2021). In CVD and diabetes-induced HFpEF models, cardiomyocyte NF-κB activation is linked to left ventricular remodeling and elevated expression of inflammatory cytokines and fibrotic factors (Hulsmans et al., 2018; Roche et al., 2015; Zuo et al., 2024). Consequently, NF-κB serves as a potential therapeutic target in HFpEF. ɑ7nAChR-mediated signaling has been shown to inhibit NF-κB activation, reducing the expression of IL-1β, IL-6, MCP-1, TNF-α, MCP-1, vascular adhesion molecule-1 (VCAM-1), and intracellular adhesion molecule 1 (ICAM-1) (Zhang et al., 2013; Saeed et al., 2005). In hypertensive rats, the ɑ7nAChR agonist, PNU-282987, suppresses inflammation and protects against left ventricular damage and aortic thickening, partly through NF-κB inhibition (Li et al., 2011). These findings indicate the role of the ɑ7nAChR in regulating NF-κB as a mechanism for benefiting heart health.

Emerging evidence suggests that inflammasomes play a critical role in HFpEF (Li et al., 2022; Cheng et al., 2023). The Nod-like receptor pyrin domain containing 3 (NLRP3) inflammasome is a critical component of cardiac immune responses (Yao et al., 2018). In murine models of HFpEF, increased NLRP3 inflammasome activation is implicated in cardiomyocyte fibrosis, cardiac necrosis, left ventricular arrhythmia, and poor survival prognosis (Cheng et al., 2023; Yang HJ. et al., 2020). Given that inhibiting NLRP3 has been shown to improve left ventricular diastolic function and reduce cardiac inflammation and fibrosis in patients with HFpEF (Yang et al., 2024; Peh et al., 2023), it has emerged as a new target in HF management (Cheng et al., 2023). Although the role of ɑ7nAChR in cardiac NLRP3 regulation has yet to be investigated, neuroinflammatory and osteoarthritis models have demonstrated that receptor activation reduces NLRP3 activity to modulate pro-inflammatory responses. Furthermore, PNU-282987 has been reported to alleviate inflammation-induced aortic cell death dependent on NLRP3 inhibition (Fu et al., 2022), which may translate to cardiovascular protection against HFpEF.

HMGB1 acts as a DAMP to drive inflammation (Yang H. et al., 2020). In response to cellular stress, DAMPs are passively released from the nucleus of damaged cells to initiate an inflammatory response (Chen et al., 2022). Binding of HMBG1 to its receptors (e.g., TLR4 and RAGE) triggers inflammation and immune cell infiltration through NF-kB upregulation (Wahid et al., 2021; Oeckinghaus et al., 2011). Myocardial biopsies from HFpEF patients have shown significant upregulation of HMBG1 and TLR4 (Delalat et al., 2025). Mover, inhibiting HMGB1 expression in cardiac tissue of HFpEF mice reduces neutrophil infiltration and improves diastolic function (Zhang XL. et al., 2022). Consistent with a protective role of ɑ7nAChR through this pathway, agonism with GTS-21 has been demonstrated to attenuate hyperoxia-induced lung inflammation by reducing HMGB1 accumulation in serum, decreasing macrophage infiltration in lung tissue, and attenuating lung injury (Sitapara et al., 2020). Conversely, genetic deletion of ɑ7nAChR appeared to upregulate HMGB1 expression through an increase in NLRP3 activity (Lu et al., 2014). Interestingly, in a murine model of inflammation-induced chronic pain, central injection of ɑ7nAChR antagonist, α-BTX, exacerbated HMGB1 and NF-κB expression (Sun et al., 2022). Given these studies support the potential of ɑ7nAChR to regulate HMGB1 release, this pathway may provide another therapeutic anti-inflammatory strategy to combat HF.

## 4 α7nAChR and the RAAS

The RAAS is a major hormonal regulator of cardiovascular physiological and is instrumental in maintaining the homeostatic balance of blood pressure and systemic vascular resistance (Ruan et al., 2024). Overactivation of the RAAS is also a central component in the pathophysiology of HF, and drugs that target the RAAS have become important pillars of HF and CVD therapy (Ichikawa et al., 2025; Singhania et al., 2020). Angiotensin II (AngII) is the principal effector of the RAAS, and is synthesized by angiotensin-converting enzyme (ACE) within the classic axis of the RAAS, and exerts its tissue-dependent effects through the AngII type 1 receptor (AT_1_R) to stimulate vasoconstriction, aldosterone release, and fluid retention in the kidneys (Ruan et al., 2024). Beyond its role in hemodynamic regulation, AngII/AT_1_R signaling is well established in promoting cardiac inflammation through upregulation of NF-κB (Muller et al., 2000), NRLP3 (Espitia-Corredor et al., 2022), and HMGB1 (Zhang et al., 2021), resulting in fibrotic, apoptotic, and oxidative damage associated with HF (Beldhuis et al., 2017; Carter et al., 2024). Thus, it stands to reason that inhibition of this pro-inflammatory signaling pathway may be key to managing pathophysiological damage in HFpEF. Despite RAAS inhibitors (i.e., ACE inhibitors (ACEi) and AT_1_R receptor blockers (ARBs) being commonly prescribed in patients with HF, the overall prognosis remains poor (Odajima et al., 2022; Kjeldsen et al., 2020).

Although the current understanding of ɑ7nAChR in RAS signaling remains limited, emerging evidence suggests that ɑ7nAChR may have a regulatory role in mitigating AngII/AT1R signaling. In neuronal cells, ɑ7nAChR plays a protective role in promoting cell survivability against AngII-induced stress (Shaw et al., 2003), while receptor against GTS-21 is shown to prevent AngII-mediated elevation of blood pressure, improve parasympathetic baroreflex control, and prevent pro-inflammatory NF-κB activation in response to AngII (Wu SJ. et al., 2021). Similarly, in a murine model of atherosclerosis, ɑ7nAChR agonist AR-R17779 reduces AT1R expression in vascular smooth muscle cells, suppressing AngII-mediated formation of atherosclerotic plaque (Hashimoto et al., 2014). This effect was also associated with a significant decrease in aortic tissue expression of IL-6, IL-1β, and pro-oxidative membrane complex NADPH oxidase (Hashimoto et al., 2014). The physiological importance of ɑ7nAChR in RAAS regulation is also observed in α7AChR^−/−^ mice, as deletion of this receptor exacerbates vascular injury in response to AngII infusion (Li et al., 2016). Importantly, this injury was alleviated in wild-type mice treated with PNU-282987, suggesting the important of ɑ7nAChR in regulation of AngII-mediated pathology (Li et al., 2016). Given that vascular injury is a major pathological phenotype of HFpEF, the ability of ɑ7nAChR to protect against AngII-induced vascular damage may be of crucial importance to its therapeutic value.

Angiotensin-converting enzyme-2 (ACE2) is a member of the counter-regulatory RAAS axis and is responsible for mitigating the deleterious actions of AngII/AT_1_R and provides cardioprotective effects (Bhushan et al., 2023; Zisman, 2005). ACE2 degrades AngII into angiotensin 1–7 [Ang (Roth et al., 2020; Norhammar et al., 2023; Savarese et al., 2022; McDonagh et al., 2021; Dini et al., 2023; Thibodeau and Drazner, 2018; Naing et al., 2019)], which acts *via* the Mas1 oncogene receptor (MasR) to oppose the actions of AngII by promoting vasodilation through endothelial nitric oxide (NO) release (Fraga-Silva et al., 2013), reducing fibrosis, and mitigating pro-oxidative and pro-inflammatory signaling. Altered ACE2 expression/activity is directly linked to the progression of heart disease (Patel et al., 2016). Mice that lack ACE2 show elevated AngII and reduced Ang (Roth et al., 2020; Norhammar et al., 2023; Savarese et al., 2022; McDonagh et al., 2021; Dini et al., 2023; Thibodeau and Drazner, 2018; Naing et al., 2019) levels as well as an enhanced cardiac susceptibility to pathological ventricular remodeling, reactive oxygenated species accumulation, and an increase in pressure-overload-induced HF (Kassiri et al., 2009; Patel et al., 2014; Zhong et al., 2010). In contrast, overexpression of ACE2 in mice or treatment of rats with recombinant ACE2 protects the heart against myocardial injury and counteracts HFpEF, demonstrating the beneficial effects of enhancing ACE2 activity (Chamsi-Pasha et al., 2014; Zhao et al., 2010). In epithelial cells, cholinergic agonist, nicotine, is observed to upregulate ACE2 expression (Maggi et al., 2021), while silencing of the ɑ7nAChR gene attenuates this effect, suggesting a potential regulatory role of ɑ7nAChR signaling in promoting ACE2 expression (Cattaruzza et al., 2020). Furthermore, GTS-21 has been observed to significantly increase the ratio of ACE2 to ACE and shifts protein levels away from AngII/AT_1_R towards Ang (Roth et al., 2020; Norhammar et al., 2023; Savarese et al., 2022; McDonagh et al., 2021; Dini et al., 2023; Thibodeau and Drazner, 2018; Naing et al., 2019)/MasR in murine models of chronic inflammation (Zhu et al., 2024). This effect has also been associated with inhibition of macrophage polarization, reduced NF-κB activation, and a decrease in IL-1β, Il-6, TNF-α, and HMGB-1 levels, suggesting a link between ɑ7nAChR and reduced pro-inflammatory signaling through enhanced RAAS regulation (Zhu et al., 2024). Collectively, these studies establish the potential regulatory role of ɑ7nAChR in RAAS signaling. Therapeutic strategies targeting ɑ7nAChR may therefore represent a promising approach to HFpEF by reducing cardiac inflammation, ventricular remodeling, and oxidative damage through coordinated suppression of ACE/AngII/AT1R and activation of ACE2/Ang (Roth et al., 2020; Norhammar et al., 2023; Savarese et al., 2022; McDonagh et al., 2021; Dini et al., 2023; Thibodeau and Drazner, 2018; Naing et al., 2019)/MasR pathway.

## 5 α7nAChR in hypertension and vascular regulation

Hypertension is the most prevalent comorbidity associated with HFpEF (Anker et al., 2021; Solomon et al., 2019). An increase in 1 mmHg systolic blood pressure >120 mmHg increases the risk of HFpEF by 3% in an acute HF setting (Styron et al., 2009). Hypertension causes a persistent pressure overload in the left ventricle, subsequently leading to left ventricular hypertrophy, fibrosis, stiffness, and eventual ventricular diastolic dysfunction (Guazzi et al., 2020; Mishra and Kass, 2021). Hypertension is also associated with sympathetic and RAAS overactivation, systemic inflammation, endothelial dysfunction, and immune cell recruitment into the myocardium, contributing to the progression of HFpEF (Levick et al., 2010). A comprehensive meta-analysis of 123 studies with over 600,000 participants has shown that a 10 mmHg reduction in blood pressure is enough to reduce the risk of HF by 28%; thus, controlling blood pressure is deemed critical to preventing the cardiovascular events of HF (Ettehad et al., 2016).

Baroreflexes are key regulators of autonomic cardiovascular stability and hemodynamic control (Clemmer et al., 2022). This system detects changes in blood pressure and relays this information to the CNS, which adjusts sympathetic autonomic outflow and RAAS activity to modulate peripheral resistance and cardiac output *via* changes in cardiac contractility, heart rate, and arterial vasoconstriction (Levick et al., 2010; Munoz-Durango et al., 2016). Baroreflex insensitivity stemming from autonomic dysfunction impairs blood pressure regulation and is a key feature in patients with resistant hypertension and HFpEF (Clemmer et al., 2022; Grassi et al., 2014). These patients exhibit a marked increase in sympathetic nerve activity, which is believed to contribute to the limited efficacy of ACEi or ARBs beyond blood pressure reduction (Georgakopoulos et al., 2011; Yaxley and Thambar, 2015). Clinical studies have shown that promoting a shift from sympathetic innervation at the baroreceptor level is favorable to improving cardiac efficiency and reducing adverse remodeling to combat HFpEF (Mazidi et al., 2017; Seravalle et al., 2019). Hypertensive models have shown that baroreflex dysfunction is closely linked with decreased tissue expression of ɑ7nAChR and elevated circulation of inflammatory mediators (Guo et al., 2017; Chen et al., 2012). Moreover, ɑ7nAChR deficiency is observed to exacerbate baroreflex dysfunction (Liu et al., 2012). Pharmacological activation of ɑ7nAChR has been demonstrated to mitigate baroreflex dysfunction and alleviate hypertension-induced end-organ damage (Li et al., 2011; Guo et al., 2017). Most importantly, vagal nerve stimulation has been shown to effectively attenuate blood pressure elevation in hypertensive rats to the same degree as the clinically prescribed ARB, Olmesartan. This effect was dependent on ɑ7nAChR, as demonstrated by its attenuation with receptor antagonist MLA (Elkholey et al., 2022).

In addition to central regulation of cardiovascular hemodynamics, the ɑ7nAChR has also been implicated in local cardiac cholinergic signaling. Positive CHRNA7 protein and ɑ7nAChR expression has been detected in cardiac tissue, localized to cardiac fibroblasts, neurons, and cardiomyocytes (Li et al., 2019; Dvorakova et al., 2005). Here, functional studies have indicated that the receptor may participate in local cholinergic regulation of the heart under specific stress conditions. In Langendorff preparations of the isolated rat heart, nicotine-induced bradycardia was blocked by the ɑ7nAChR antagonist, ɑ-BTX (Ji et al., 2002). Other studies have demonstrated that receptor antagonist, MLA, attenuates cardiac sympathetic pressor responses with no effect on bradycardia responses (Li et al., 2009). These results are consistent with α7AChR knockout models, showing that receptor deletion has no effect on baroreflex (Franceschini et al., 2000) and vagal simulation-induced bradycardia (Deck et al., 2005). A slight reduction in heart rate has been observed in mice lacking α7AChR, only following cardiac exposure to high ACh concentrations (Targosova et al., 2024), suggesting that cardiac α7AChR plays a direct role in local cholinergic overstimulation. This is supported by studies in Schwann cells (key component of neuromuscular junction), which show that α7AChR reduces ACh release following inhibition of cholinesterases (Petrov et al., 2014). Furthermore, in rats with myocardial infarction-induced chronic HF, AChE inhibitor, donepezil, significantly enhanced vagal tone, improved heart rate and cardiac function, as well as reducing cardiac remodeling, fibrosis, and inflammation The role of ɑ7nAChR in cholinergic regulation of the heart and anti-inflammatory signaling has also been linked to cholinesterases, such as AChE and BChE. While AChE is classically associated with synaptic ACh hydrolysis, BChE is abundant in non-neuronal tissue and contributes substantially to ACh degeneration in the heart (Dingova et al., 2025). Reduced ACh availability has been associated with early onset CVDs, contributing to increased cardiac mortality and impaired myocardial regeneration (Liu et al., 2024). These alterations are linked to autonomic cardiovascular dysregulation, enhanced AngII/AT_1_R signaling (Xu et al., 2018; Liu et al., 2011), and myocardial oxidative and inflammatory damage. AChE and BChE are both expressed in the atrial and ventricles (Dingova et al., 2025; Kilianova et al., 2020), and elevated circulation of these enzymes has been reported in major HF risk factors, including obesity and type II diabetes (Villeda-Gonzalez et al., 2024). AChE and BChE activity are key determinants of local Ach bioavailability, with BChE contributing significantly to ACh hydrolysis in the cardiac tissue. Thus, these enzymes are suggested to play a fundamental role in ɑ7nAChR signaling (Dingova et al., 2025). At the cellular level, suppression of AChR and BChR mRNA increases ɑ7nAChR expression, leading to an enhanced anti-inflammatory response (Nadorp and Soreq, 2015). Furthermore, ɑ7nAChR activation triggers intracellular calcium ion influx which inhibits AChE expression in macrophages, suggesting the presence of a feedback mechanism regulating local cholinergic tone (Liu et al., 2020). This potential feedback mechanism is also supported by investigations at the neuromuscular junction where ɑ7nAChR activation inhibits further release of ACh (Petrov et al., 2025).

Capillary rarefaction refers to a reduction in capillary density and shows a close association with the functional decline of the left ventricle in HFpEF patients (Mohammed et al., 2015). Multiple studies in hypertensive modes show diminished left ventricle capillary density, and is strongly associated with inflammation and RAAS overactivation (Gonzalez et al., 2015; Ihori et al., 2016; Yazawa et al., 2011). An increase in systemic circulation of pro-inflammatory cytokines and AngII can lead to heart capillary rarefaction through endothelial dysfunction and a subsequent reduction in NO and cyclic guanosine monophosphate bioavailability, which inhibits vasodilation and favors oxidative and fibrotic cardiac damage (Franssen et al., 2016; Tam et al., 2017). ɑ7nAChR expression on vascular cells has been established (Wada et al., 2007; Conti-Fine et al., 2000; Heeschen et al., 2002)regulate cellular proliferation, inflammation, oxidative stress, and increase cell survival (Vieira-Alves et al., 2020; Liu L. et al., 2017). In cultured endothelial cells, ɑ7nAChR agonist, PNU-282987, has been observed to increase endothelial NO synthase expression/activity, resulting in the subsequent production of NO (Wu et al., 2023). This was prevented by the antagonist, MLA. Neuronal cells have also demonstrated ɑ7nAChR-dependent coupling of NO synthase to promote NO production (Haberberger et al., 2003). This increase in NO bioavailability may be associated with enhanced Sirtuin-1 production. Sirtuin-1 is a protein deacetylase known to have a beneficial role in cardiovascular health through increased vascular eNOS expression and increased cardiac capillary density (Askin et al., 2020; Xia et al., 2013), for which PNU-282987 has been shown to promote in isolated mouse vascular cells, leading to the prevention of oxidative stress and proliferative arrest (Li et al., 2016). Moreover, nicotine is observed to enhance capillary density in the ischemic heart of myocardial infarction-induced rats, which was inhibited by both MLA and α-BTX (Li and Wang, 2006). Since endothelial dysfunction and impaired NO signaling are pathological phenotypes of HFpEF patients, these studies suggest that ɑ7nAChR may participate in vasoprotective effects that improve vascular tone and capillary formation, underscoring it as a promising therapeutic target to restore cardiovascular regulation in HFpEF.

## 6 α7nAChR in metabolic regulation

Metabolic dysfunction is a major contributor to HFpEF. Unlike HFrEF, HFpEF is strongly associated with metabolic disorders such as obesity and Type II diabetes (Jackson et al., 2022; Pandey et al., 2017; Prausmuller et al., 2023). These comorbidities can contribute to the overlap between chronic inflammation, sympathetic nervous system and RAAS activation, endothelial dysfunction and hypertension, leading to pathogenic myocardial remodeling and stiffness (Borlaug et al., 2023; van Dalen et al., 2025). While traditional HF therapies, including RAAS inhibitors and mineralocorticoid antagonists, show limited benefit in metabolic comorbidities linked to HFpEF (Borlaug et al., 2023), recent clinical investigations targeting these pathways, such as SGLT2 inhibitors (Solomon et al., 2021; Anker et al., 2021), statins (Alehagen et al., 2015; Schiattarella et al., 2021), and weight loss (Savji et al., 2018; Kitzman et al., 2016) are linked with a significant reduction in HF hospitalization and mortality, underscoring the importance of metabolic regulation in HFpEF management.

Recent studies highlight a protective role of ɑ7nAChR in metabolic disorders. In high-fat-fed obese mice and obese patients, ɑ7nAChR mRNA and protein levels are markedly reduced compared with normal-weight controls (Cancello et al., 2012; Li et al., 2018). Receptor deficiency in high-fat animals is also associated with severe chronic low-grade inflammation (Souza et al., 2019), whereas activation downregulates inflammatory gene expression and limits adipose macrophage infiltration (Chang et al., 2019; Pavlov and Tracey, 2012). A decrease in adipose tissue inflammation is strongly associated with improvements in hypertension and vascular dysfunction (Chait and den Hartigh, 2020; Jin et al., 2023). In AngII-accelerated atherosclerotic mice on a high-fat diet, the ɑ7nAChR agonist, AR-R17779, lowered blood pressure, reduced circulating lipids, IL-6, and TNF, and improved survival (Hashimoto et al., 2014). Additionally, ɑ7nAChR activation by GTS-21 has been shown to enhance glucagon-like peptide-1 (GLP-1) activity, a hormone involved in glucose regulation, appetite suppression, and cardiovascular inflammation (Xie et al., 2020). Providing some insight into a mechanistic link and outcomes, early clinical and experimental data suggest GLP-1 activation provides protective cardiovascular effects against HFpEF (Cimino et al., 2024; Butler et al., 2023). Collectively, these studies suggest that ɑ7nAChR activation could provide beneficial effects in HFpEF by improving metabolic regulation and attenuating associated inflammation.

## 7 Perspectives and conclusion

Over time, evidence has mounted to build a compelling case that HFpEF is a multifactorial disorder hallmarked by several mechanisms, including inflammation, endothelial dysfunction, potentiated by hypertension and metabolic disorders. As such, new therapies that are effective across this broad spectrum of pathologies are required. This review highlights substantial evidence supporting the physiological importance and therapeutic potential of α7nAChR in HF and HFpEF management. In cases where, ACEi, ARBs, or other antihypertensive agents only partially benefit, ɑ7nAChR activation may offer complementary cardioprotective effects against HFpEF through its direct or indirect regulation of systemic and local cardiovascular inflammation through NF-κB/NLRP3/HMGB1 suppression, changes in RAAS classical and alternative axis components, improved baroreflex and vascular signaling, and modulation to metabolic and neural pathways to mitigate cardiac damage and alleviate comorbidities associated with HF, such as obesity, dyslipidemia, and hypertension. Future studies are needed to identify the most effective and well-tolerated α7nAChR agonist and to determine the clinical significance of targeting this receptor in patients. Additionally, studies should seek to further characterize the physiological importance of central and peripheral α7nAChR in cardiovascular regulation, as well as the therapeutic efficacy between orthostatic and PAM receptor agonism, given their differences in receptor desensitization, tissue specificity, and reliance of endogenous ACh cholinergic signaling.

## References

[B1] Abot A. Cani P. D. Knauf C. (2018). Impact of intestinal peptides on the enteric nervous system: novel approaches to control glucose metabolism and food intake. Front. Endocrinol. (Lausanne) 9, 328. 10.3389/fendo.2018.00328 29988396 PMC6023997

[B2] Aghajanian H. Kimura T. Rurik J. G. Hancock A. S. Leibowitz M. S. Li L. (2019). Targeting cardiac fibrosis with engineered T cells. Nature 573 (7774), 430–433. 10.1038/s41586-019-1546-z 31511695 PMC6752964

[B3] Al-Sharea A. Lee M. K. S. Whillas A. Flynn M. C. Chin-Dusting J. Murphy A. J. (2017). Nicotinic acetylcholine receptor alpha 7 stimulation dampens splenic myelopoiesis and inhibits atherogenesis in Apoe(-/-) mice. Atherosclerosis 265, 47–53. 10.1016/j.atherosclerosis.2017.08.010 28858686

[B4] Alehagen U. Benson L. Edner M. Dahlstrom U. Lund L. H. (2015). Association between use of statins and outcomes in heart failure with reduced ejection fraction: prospective propensity score matched cohort study of 21 864 patients in the Swedish heart failure registry. Circ. Heart Fail 8 (2), 252–260. 10.1161/CIRCHEARTFAILURE.114.001730 25575580

[B5] Anker S. D. Butler J. Filippatos G. Ferreira J. P. Bocchi E. Bohm M. (2021). Empagliflozin in heart failure with a preserved ejection fraction. N. Engl. J. Med. 385 (16), 1451–1461. 10.1056/NEJMoa2107038 34449189

[B6] Arunrungvichian K. Vajragupta O. Hayakawa Y. Pongrakhananon V. (2023). Targeting alpha7 nicotinic acetylcholine receptors in lung cancer: insights, challenges, and therapeutic strategies. ACS Pharmacol. Transl. Sci. 7 (1), 28–41. 10.1021/acsptsci.3c00138 38230275 PMC10789132

[B7] Askin L. Tibilli H. Tanriverdi O. Turkmen S. (2020). The relationship between coronary artery disease and SIRT1 protein. North Clin. Istanb. 7 (6), 631–635. 10.14744/nci.2020.31391 33381707 PMC7754863

[B8] Aune D. Schlesinger S. Leitzmann M. F. Tonstad S. Norat T. Riboli E. (2021). Physical activity and the risk of heart failure: a systematic review and dose–response meta-analysis of prospective studies. Eur. J. Epidemiol. 36, 367–381. 10.1007/s10654-020-00693-6 33331992 PMC8076120

[B9] Bali Z. K. Nagy L. V. Budai D. Hernádi I. (2019). Facilitation and inhibition of firing activity and N-methyl-D-aspartate-evoked responses of CA1 hippocampal pyramidal cells by alpha7 nicotinic acetylcholine receptor selective compounds *in vivo* . Sci. Rep. 9 (1), 9324. 10.1038/s41598-019-45796-7 31249369 PMC6597544

[B10] Baffour P. K. Jahangiry L. Jain S. Sen A. Aune D. (2024). Blood pressure, hypertension, and the risk of heart failure: a systematic review and meta-analysis of cohort studies. Eur. J. Prev. Cardiol. 31 (5), 529–556. 10.1093/eurjpc/zwad344 37939784

[B11] Bagdas D. Wilkerson J. L. Kulkarni A. Toma W. AlSharari S. Gul Z. (2016). The alpha7 nicotinic receptor dual allosteric agonist and positive allosteric modulator GAT107 reverses nociception in mouse models of inflammatory and neuropathic pain. Br. J. Pharmacol. 173 (16), 2506–2520. 10.1111/bph.13528 27243753 PMC4959951

[B12] Bahit M. C. Kochar A. Granger C. B. (2018). Post-myocardial infarction heart failure. JACC Heart Failure 6 (3), 179–186. 10.1016/j.jchf.2017.09.015 29496021

[B13] Bairey Merz C. N. Pepine C. J. Shimokawa H. Berry C. (2020). Treatment of coronary microvascular dysfunction. Cardiovasc Res. 116 (4), 856–870. 10.1093/cvr/cvaa006 32087007 PMC7061279

[B14] Barrantes G. E. Westwick J. Wonnacott S. (1994). Nicotinic acetylcholine receptors in primary cultures of hippocampal neurons: pharmacology and ca++ permeability. Biochem. Soc. Trans. 22 (3), 294S. 10.1042/bst022294s 7821553

[B15] Bashier A. Bin Hussain A. Abdelgadir E. Alawadi F. Sabbour H. Chilton R. (2019). Consensus recommendations for management of patients with type 2 diabetes mellitus and cardiovascular diseases. Diabetol. Metab. Syndr. 11, 80. 10.1186/s13098-019-0476-0 31572499 PMC6761728

[B16] Bauersachs J. Kato E. T. Rangaswami J. (2025). Interconnected pathways and emerging therapies in chronic kidney disease and heart failure: a comprehensive review. ESC. Heart Fail. 12 (5), 3226–3249. 10.1002/ehf2.15345 40533425 PMC12450771

[B17] Beldhuis I. E. Streng K. W. Ter Maaten J. M. Voors A. A. van der Meer P. Rossignol P. (2017). Renin–angiotensin system inhibition, worsening renal function, and outcome in heart failure patients with reduced and preserved ejection fraction: a meta-analysis of published study data. Circ. Heart Fail. 10 (2), e003588. 10.1161/CIRCHEARTFAILURE.116.003588 28209765

[B18] Bhagat A. Shrestha P. Kleinerman E. S. (2022). The innate immune system in cardiovascular diseases and its role in doxorubicin-induced cardiotoxicity. Int. J. Mol. Sci. 23 (23), 14649. 10.3390/ijms232314649 36498974 PMC9739741

[B19] Bhushan S. Xiao Z. Gao K. Mao L. Chen J. Ping W. (2023). Role and interaction between ACE1, ACE2 and their related genes in cardiovascular disorders. Curr. Probl. Cardiol. 48 (8), 101162. 10.1016/j.cpcardiol.2022.101162 35245599

[B20] Billingsley H. E. Hummel S. L. Carbone S. (2020). The role of diet and nutrition in heart failure: a state-of-the-art narrative review. Prog. Cardiovas. Dis. 63 (5), 538–551. 10.1016/j.pcad.2020.08.004 32798501 PMC7686142

[B21] Blanton R. M. Carrillo-Salinas F. J. Alcaide P. (2019). T-cell recruitment to the heart: friendly guests or unwelcome visitors? Am. J. Physiol. Heart Circ. Physiol. 317 (1), H124–H140. 10.1152/ajpheart.00028.2019 31074651 PMC6692732

[B22] Bodnar A. L. Cortes-Burgos L. A. Cook K. K. Dinh D. M. Groppi V. E. Hajos M. (2005). Discovery and structure− activity relationship of quinuclidine benzamides as agonists of α7 nicotinic acetylcholine receptors. J. Med. Chem. 48 (4), 905–908. 10.1021/jm049363q 15715459

[B23] Borlaug B. A. Jensen M. D. Kitzman D. W. Lam C. S. P. Obokata M. Rider O. J. (2023). Obesity and heart failure with preserved ejection fraction: new insights and pathophysiological targets. Cardiovasc Res. 118 (18), 3434–3450. 10.1093/cvr/cvac120 35880317 PMC10202444

[B24] Borovikova L. V. Ivanova S. Zhang M. Yang H. Botchkina G. I. Watkins L. R. (2000). Vagus nerve stimulation attenuates the systemic inflammatory response to endotoxin. Nature 405 (6785), 458–462. 10.1038/35013070 10839541

[B25] Boulet J. Sridhar V. S. Bouabdallaoui N. Tardif J. C. White M. (2024). Inflammation in heart failure: pathophysiology and therapeutic strategies. Inflamm. Res. 73 (5), 709–723. 10.1007/s00011-023-01845-6 38546848 PMC11058911

[B26] Brar S. Goli R. Barrios J. P. Blaha M. J. Kianoush S. Pletcher M. J. (2025). Association of extracoronary calcification and incident heart failure in the Multiethnic Study of Atherosclerosis (MESA). Heart Fail. 13 (5), 740–751. 10.1016/j.jchf.2024.12.007 40146086 PMC12510633

[B27] Broide R. S. Winzer-Serhan U. H. Chen Y. Leslie F. M. (2019). Distribution of alpha7 nicotinic acetylcholine receptor subunit mRNA in the developing mouse. Front. Neuroanat. 13, 76. 10.3389/fnana.2019.00076 31447654 PMC6691102

[B28] Butler J. Abildstrom S. Z. Borlaug B. A. Davies M. J. Kitzman D. W. Petrie M. C. (2023). Semaglutide in patients with obesity and heart failure across mildly reduced or preserved ejection fraction. J. Am. Coll. Cardiol. 82 (22), 2087–2096. 10.1016/j.jacc.2023.09.811 37993201 PMC11185158

[B29] Cancello R. Zulian A. Maestrini S. Mencarelli M. Della Barba A. Invitti C. (2012). The nicotinic acetylcholine receptor alpha7 in subcutaneous mature adipocytes: downregulation in human obesity and modulation by diet-induced weight loss. Int. J. Obes. (Lond). 36 (12), 1552–1557. 10.1038/ijo.2011.275 22270376

[B30] Cantero-Navarro E. Fernandez-Fernandez B. Ramos A. M. Rayego-Mateos S. Rodrigues-Diez R. R. Sanchez-Nino M. D. (2021). Renin-angiotensin system and inflammation update. Mol. Cell Endocrinol. 529, 111254. 10.1016/j.mce.2021.111254 33798633

[B31] Cao Y. Sun J. Wang X. Zhang X. Tian H. Huang L. (2024). The double-edged nature of nicotine: toxicities and therapeutic potentials. Front. Pharmacol. 15, 1427314. 10.3389/fphar.2024.1427314 39206262 PMC11350241

[B32] Carter K. Shah E. Waite J. Rana D. Zhao Z. Q. (2024). Pathophysiology of Angiotensin II-mediated hypertension, cardiac hypertrophy, and failure: a perspective from macrophages. Cells 13 (23), 2001. 10.3390/cells13232001 39682749 PMC11640308

[B33] Cattaruzza M. S. Gorini G. Bosetti C. Boffi R. Lugo A. Veronese C. (2020). Covid-19 and the role of smoking: the protocol of the multicentric prospective study COSMO-IT (COVID19 and Smoking in Italy). Acta Biomed. 91 (3), e2020062. 10.23750/abm.v91i3.10373 32921714 PMC7716969

[B34] Chait A. den Hartigh L. J. (2020). Adipose tissue distribution, inflammation and its metabolic consequences, including diabetes and cardiovascular disease. Front. Cardiovasc Med. 7, 22. 10.3389/fcvm.2020.00022 32158768 PMC7052117

[B35] Chamsi-Pasha M. A. Shao Z. Tang W. H. (2014). Angiotensin-converting enzyme 2 as a therapeutic target for heart failure. Curr. Heart Fail Rep. 11 (1), 58–63. 10.1007/s11897-013-0178-0 24293035 PMC3944399

[B36] Chang E. H. Chavan S. S. Pavlov V. A. (2019). Cholinergic control of inflammation, metabolic dysfunction, and cognitive impairment in obesity-associated disorders: mechanisms and novel therapeutic opportunities. Front. Neurosci. 13, 263. 10.3389/fnins.2019.00263 31024226 PMC6460483

[B37] Chauhan A. Islam A. U. Prakash H. Singh S. (2022). Phytochemicals targeting NF-kappaB signaling: potential anti-cancer interventions. J. Pharm. Anal. 12 (3), 394–405. 10.1016/j.jpha.2021.07.002 35811622 PMC9257438

[B38] Chen J. K. Zhao T. Ni M. Li D. J. Tao X. Shen F. M. (2012). Downregulation of alpha7 nicotinic acetylcholine receptor in two-kidney one-clip hypertensive rats. BMC Cardiovasc Disord. 12, 38. 10.1186/1471-2261-12-38 22682236 PMC3507811

[B39] Chen R. Kang R. Tang D. (2022). The mechanism of HMGB1 secretion and release. Exp. Mol. Med. 54 (2), 91–102. 10.1038/s12276-022-00736-w 35217834 PMC8894452

[B40] Cheng X. Zhao H. Wen X. Li G. Guo S. Zhang D. (2023). NLRP3-inflammasome inhibition by MCC950 attenuates cardiac and pulmonary artery remodelling in heart failure with preserved ejection fraction. Life Sci. 333, 122185. 10.1016/j.lfs.2023.122185 37858713

[B41] Chioncel O. Ambrosy A. P. (2020). Hyperglycemia and outcomes in acute heart failure–a bittersweet relationship. Int. J. Cardiol. 300, 196–197. 10.1016/j.ijcard.2019.09.030 31776008

[B42] Chirinos J. A. Orlenko A. Zhao L. Basso M. D. Cvijic M. E. Li Z. (2020). Multiple plasma biomarkers for risk stratification in patients with heart failure and preserved ejection fraction. J. Am. Coll. Cardiol. 75 (11), 1281–1295. 10.1016/j.jacc.2019.12.069 32192654 PMC7147356

[B43] Cimino G. Vaduganathan M. Lombardi C. M. Pagnesi M. Vizzardi E. Tomasoni D. (2024). Obesity, heart failure with preserved ejection fraction, and the role of glucagon-like peptide-1 receptor agonists. Esc. Heart Fail 11 (2), 649–661. 10.1002/ehf2.14560 38093506 PMC10966224

[B44] Clemmer J. S. Pruett W. A. (2022). Modeling the physiological roles of the heart and kidney in heart failure with preserved ejection fraction during baroreflex activation therapy. Am. J. Physiol. Heart Circ. Physiol. 323 (3), H597–H607. 10.1152/ajpheart.00329.2022 35984764 PMC9467477

[B45] Conraads V. M. Metra M. Kamp O. De Keulenaer G. W. Pieske B. Zamorano J. (2012). Effects of the long-term administration of nebivolol on the clinical symptoms, exercise capacity, and left ventricular function of patients with diastolic dysfunction: results of the ELANDD study. Eur. J. Heart Fail 14 (2), 219–225. 10.1093/eurjhf/hfr161 22147202

[B46] Conti-Fine B. M. Navaneetham D. Lei S. Maus A. D. (2000). Neuronal nicotinic receptors in non-neuronal cells: new mediators of tobacco toxicity? Eur. J. Pharmacol. 393 (1-3), 279–294. 10.1016/s0014-2999(00)00036-4 10771024

[B47] Dacosta C. J. Free C. R. Sine S. M. (2015). Stoichiometry for α-bungarotoxin block of α7 acetylcholine receptors. Nat. Communications 6 (1), 8057. 10.1038/ncomms9057 26282895 PMC4544739

[B48] Daou D. Gillette T. G. Hill J. A. (2023). Inflammatory mechanisms in heart failure with preserved ejection fraction. Physiol. (Bethesda) 38 (5), 0. 10.1152/physiol.00004.2023 37013947 PMC10396273

[B49] Deck J. Bibevski S. Gnecchi-Ruscone T. Bellina V. Montano N. Dunlap M. E. (2005). Alpha7-nicotinic acetylcholine receptor subunit is not required for parasympathetic control of the heart in the mouse. Physiol. Genomics 22 (1), 86–92. 10.1152/physiolgenomics.00085.2004 15797970

[B50] Delalat S. Sultana I. Osman H. Sieme M. Zhazykbayeva S. Herwig M. (2025). Dysregulated inflammation, oxidative stress, and protein quality control in diabetic HFpEF: unraveling mechanisms and therapeutic targets. Cardiovasc Diabetol. 24 (1), 211. 10.1186/s12933-025-02734-4 40369521 PMC12080046

[B51] Dingova D. Kucera M. Hodbod T. Fischmeister R. Krejci E. Hrabovska A. (2025). Cardiac acetylcholinesterase and butyrylcholinesterase have distinct localization and function. Am. J. Physiol. Heart Circ. Physiol. 328 (3), H526–H542. 10.1152/ajpheart.00672.2024 39836467

[B52] Dini F. L. Pugliese N. R. Ameri P. Attanasio U. Badagliacca R. Correale M. (2023). Right ventricular failure in left heart disease: from pathophysiology to clinical manifestations and prognosis. Heart Fail. Rev. 28 (4), 757–766. 10.1007/s10741-022-10282-2 36284079 PMC9596338

[B53] DuBrock H. M. AbouEzzeddine O. F. Redfield M. M. (2018). High-sensitivity C-reactive protein in heart failure with preserved ejection fraction. PLoS One 13 (8), e0201836. 10.1371/journal.pone.0201836 30114262 PMC6095520

[B54] Dvorakova M. Lips K. S. Bruggmann D. Slavikova J. Kuncova J. Kummer W. (2005). Developmental changes in the expression of nicotinic acetylcholine receptor alpha-subunits in the rat heart. Cell Tissue Res. 319 (2), 201–209. 10.1007/s00441-004-1008-1 15549397

[B55] Elkholey K. Niewiadomska M. Morris L. Whyte S. Houser J. Humphrey M. B. (2022). Transcutaneous vagus nerve stimulation ameliorates the phenotype of heart failure with preserved ejection fraction through its anti-inflammatory effects. Circ. Heart Fail 15 (8), e009288. 10.1161/CIRCHEARTFAILURE.122.009288 35862007 PMC9388556

[B56] Espitia-Corredor J. A. Boza P. Espinoza-Perez C. Lillo J. M. Rimassa-Tare C. Machuca V. (2022). Angiotensin II triggers NLRP3 inflammasome activation by a Ca(2+) signaling-dependent pathway in rat cardiac fibroblast Ang-II by a Ca(2+)-Dependent mechanism triggers NLRP3 inflammasome in *cf* . Inflammation 45 (6), 2498–2512. 10.1007/s10753-022-01707-z 35867264

[B57] Ettehad D. Emdin C. A. Kiran A. Anderson S. G. Callender T. Emberson J. (2016). Blood pressure lowering for prevention of cardiovascular disease and death: a systematic review and meta-analysis. Lancet 387 (10022), 957–967. 10.1016/S0140-6736(15)01225-8 26724178

[B58] Fan H. Gu R. Wei D. (2014). “The α7 nAChR selective agonists as drug candidates for Alzheimer’s disease,” in Advance in structural bioinformatics. Editor Wei, D., Xu, Q., Zhao, T., Dai, H. (Dordrecht: Springer) 827, 353–365. 10.1007/978-94-017-9245-5_21 25387975

[B59] Fraga-Silva R. A. Costa-Fraga F. P. Murca T. M. Moraes P. L. Martins Lima A. Lautner R. Q. (2013). Angiotensin-converting enzyme 2 activation improves endothelial function. Hypertension 61 (6), 1233–1238. 10.1161/HYPERTENSIONAHA.111.00627 23608648 PMC3733257

[B60] Franceschini D. Orr-Urtreger A. Yu W. Mackey L. Y. Bond R. A. Armstrong D. (2000). Altered baroreflex responses in alpha7 deficient mice. Behav. Brain Res. 113 (1-2), 3–10. 10.1016/s0166-4328(00)00195-9 10942027

[B61] Franssen C. Chen S. Unger A. Korkmaz H. I. De Keulenaer G. W. Tschope C. (2016). Myocardial microvascular inflammatory endothelial activation in heart failure with preserved ejection fraction. JACC Heart Fail 4 (4), 312–324. 10.1016/j.jchf.2015.10.007 26682792

[B62] Fu H. Shen Q. R. Zhao Y. Ni M. Zhou C. C. Chen J. K. (2022). Activating alpha7nAChR ameliorates abdominal aortic aneurysm through inhibiting pyroptosis mediated by NLRP3 inflammasome. Acta Pharmacol. Sin. 43 (10), 2585–2595. 10.1038/s41401-022-00876-9 35217818 PMC9525652

[B63] Fujii T. Mashimo M. Moriwaki Y. Misawa H. Ono S. Horiguchi K. (2017). Expression and function of the cholinergic system in immune cells. Front. Immunol. 8, 1085. 10.3389/fimmu.2017.01085 28932225 PMC5592202

[B64] Garg B. K. Loring R. H. (2019). GTS-21 has cell-specific anti-inflammatory effects independent of alpha7 nicotinic acetylcholine receptors. PLoS One 14 (4), e0214942. 10.1371/journal.pone.0214942 30947238 PMC6448884

[B65] Gauthier A. G. Wu J. Lin M. Sitapara R. Kulkarni A. Thakur G. A. (2021). The positive allosteric modulation of alpha7-Nicotinic cholinergic receptors by GAT107 increases bacterial lung clearance in hyperoxic mice by decreasing oxidative stress in macrophages. Antioxidants (Basel) 10 (1), 135. 10.3390/antiox10010135 33477969 PMC7835977

[B66] Georgakopoulos D. Little W. C. Abraham W. T. Weaver F. A. Zile M. R. (2011). Chronic baroreflex activation: a potential therapeutic approach to heart failure with preserved ejection fraction. J. Card. Fail 17 (2), 167–178. 10.1016/j.cardfail.2010.09.004 21300307

[B67] Glezeva N. Baugh J. A. (2014). Role of inflammation in the pathogenesis of heart failure with preserved ejection fraction and its potential as a therapeutic target. Heart Fail Rev. 19 (5), 681–694. 10.1007/s10741-013-9405-8 24005868

[B68] Gonzalez G. E. Rhaleb N. E. D'Ambrosio M. A. Nakagawa P. Liu Y. Leung P. (2015). Deletion of interleukin-6 prevents cardiac inflammation, fibrosis and dysfunction without affecting blood pressure in angiotensin II-high salt-induced hypertension. J. Hypertens. 33 (1), 144–152. 10.1097/HJH.0000000000000358 25304471 PMC4256143

[B69] Grandi A. Zini I. Flammini L. Cantoni A. M. Vivo V. Ballabeni V. (2017). alpha(7) nicotinic agonist AR-R17779 protects mice against 2,4,6-Trinitrobenzene sulfonic acid-induced colitis in a spleen-dependent way. Front. Pharmacol. 8, 809. 10.3389/fphar.2017.00809 29167641 PMC5682330

[B70] Grassi G. Seravalle G. Brambilla G. Pini C. Alimento M. Facchetti R. (2014). Marked sympathetic activation and baroreflex dysfunction in true resistant hypertension. Int. J. Cardiol. 177 (3), 1020–1025. 10.1016/j.ijcard.2014.09.138 25449517

[B71] Guazzi M. Ghio S. Adir Y. (2020). Pulmonary hypertension in HFpEF and HFrEF: JACC review topic of the week. J. Am. Coll. Cardiol. 76 (9), 1102–1111. 10.1016/j.jacc.2020.06.069 32854845

[B72] Guo J. M. Zhang L. Niu X. C. Shu H. Wang L. Su D. F. (2017). Involvement of arterial baroreflex and nicotinic acetylcholine receptor alpha7 subunit pathway in the protection of metformin against stroke in stroke-prone spontaneously hypertensive rats. Eur. J. Pharmacol. 798, 1–8. 10.1016/j.ejphar.2017.01.035 28132912

[B73] Haberberger R. V. Henrich M. Lips K. S. Kummer W. (2003). Nicotinic receptor alpha 7-subunits are coupled to the stimulation of nitric oxide synthase in rat dorsal root ganglion neurons. Histochem Cell Biol. 120 (3), 173–181. 10.1007/s00418-003-0550-3 12898272

[B74] Hammarlund M. E. Darsalia V. Mjörnstedt F. Pattanaik B. Mallard C. Rocha-Ferreira E. (2021). The selective alpha7 nicotinic acetylcholine receptor agonist AR-R17779 does not affect ischemia–reperfusion brain injury in mice. Biosci. Rep. 41 (6), BSR20210736. 10.1042/BSR20210736 34008839 PMC8200656

[B75] Hartupee J. Mann D. L. (2017). Neurohormonal activation in heart failure with reduced ejection fraction. Nat. Rev. Cardiol. 14 (1), 30–38. 10.1038/nrcardio.2016.163 27708278 PMC5286912

[B76] Hashimoto T. Ichiki T. Watanabe A. Hurt-Camejo E. Michaelsson E. Ikeda J. (2014). Stimulation of alpha7 nicotinic acetylcholine receptor by AR-R17779 suppresses atherosclerosis and aortic aneurysm formation in apolipoprotein E-deficient mice. Vasc. Pharmacol. 61 (2-3), 49–55. 10.1016/j.vph.2014.03.006 24685818

[B77] Heeschen C. Weis M. Aicher A. Dimmeler S. Cooke J. P. (2002). A novel angiogenic pathway mediated by non-neuronal nicotinic acetylcholine receptors. J. Clin. Invest 110 (4), 527–536. 10.1172/JCI14676 12189247 PMC150415

[B78] Hoogland I. C. M. Yik J. Westhoff D. Engelen-Lee J. Y. Valls Seron M. Man W. K. (2022). Microglial cell response in alpha7 nicotinic acetylcholine receptor-deficient mice after systemic infection with *Escherichia coli* . J. Neuroinflammation 19 (1), 94. 10.1186/s12974-022-02452-8 35413868 PMC9006549

[B79] Huang Y. Zhang K. Liu M. Su J. Qin X. Wang X. (2021). An herbal preparation ameliorates heart failure with preserved ejection fraction by alleviating microvascular endothelial inflammation and activating NO-cGMP-PKG pathway. Phytomedicine 91, 153633. 10.1016/j.phymed.2021.153633 34320423

[B80] Hulsmans M. Sager H. B. Roh J. D. Valero-Munoz M. Houstis N. E. Iwamoto Y. (2018). Cardiac macrophages promote diastolic dysfunction. J. Exp. Med. 215 (2), 423–440. 10.1084/jem.20171274 29339450 PMC5789416

[B81] Hurst R. S. Hajos M. Raggenbass M. Wall T. M. Higdon N. R. Lawson J. A. (2005). A novel positive allosteric modulator of the alpha7 neuronal nicotinic acetylcholine receptor: *in vitro* and *in vivo* characterization. J. Neurosci. 25 (17), 4396–4405. 10.1523/JNEUROSCI.5269-04.2005 15858066 PMC6725110

[B82] Huston J. M. Ochani M. Rosas-Ballina M. Liao H. Ochani K. Pavlov V. A. (2006). Splenectomy inactivates the cholinergic antiinflammatory pathway during lethal endotoxemia and polymicrobial sepsis. J. Exp. Med. 203 (7), 1623–1628. 10.1084/jem.20052362 16785311 PMC2118357

[B83] Ichikawa D. Kawarazaki W. Saka S. Kanaoka T. Ohnishi H. Arima H. (2025). Efficacy of renin-angiotensin system inhibitors, calcium channel blockers, and diuretics in hypertensive patients with diabetes: subgroup analysis based on albuminuria in a systematic review and meta-analysis. Hypertens. Res. 48 (6), 1880–1890. 10.1038/s41440-025-02146-7 39953235

[B84] Ihori H. Nozawa T. Sobajima M. Shida T. Fukui Y. Fujii N. (2016). Waon therapy attenuates cardiac hypertrophy and promotes myocardial capillary growth in hypertensive rats: a comparative study with fluvastatin. Heart Vessels 31 (8), 1361–1369. 10.1007/s00380-015-0779-5 26686369

[B85] Jackson A. M. Rorth R. Liu J. Kristensen S. L. Anand I. S. Claggett B. L. (2022). Diabetes and pre-diabetes in patients with heart failure and preserved ejection fraction. Eur. J. Heart Fail 24 (3), 497–509. 10.1002/ejhf.2403 34918855 PMC9542636

[B86] Ji S. Tosaka T. Whitfield B. H. Katchman A. N. Kandil A. Knollmann B. C. (2002). Differential rate responses to nicotine in rat heart: evidence for two classes of nicotinic receptors. J. Pharmacol. Exp. Ther. 301 (3), 893–899. 10.1124/jpet.301.3.893 12023516

[B87] Jiang Y. Ma H. Wang X. Wang Z. Yang Y. Li L. (2020). Protective effect of the alpha7 nicotinic receptor agonist PNU-282987 on dopaminergic neurons against 6-Hydroxydopamine, regulating anti-neuroinflammatory and the immune balance pathways in rat. Front. Aging Neurosci. 12, 606927. 10.3389/fnagi.2020.606927 33568987 PMC7868536

[B88] Jin A. H. Vetter I. Dutertre S. Abraham N. Emidio N. B. Inserra M. (2014). MrIC, a novel alpha-conotoxin agonist in the presence of PNU at endogenous alpha7 nicotinic acetylcholine receptors. Biochemistry 53 (1), 1–3. 10.1021/bi400882s 24351107

[B89] Jin X. Qiu T. Li L. Yu R. Chen X. Li C. (2023). Pathophysiology of obesity and its associated diseases. Acta Pharm. Sin. B 13 (6), 2403–2424. 10.1016/j.apsb.2023.01.012 37425065 PMC10326265

[B90] Kalappa B. I. Uteshev V. V. (2013). The dual effect of PNU-120596 on α7 nicotinic acetylcholine receptor channels. Eur. J. Pharmacol. 718 (1-3), 226–234. 10.1016/j.ejphar.2013.08.027 24036349 PMC3839061

[B91] Kallikourdis M. Martini E. Carullo P. Sardi C. Roselli G. Greco C. M. (2017). T cell costimulation blockade blunts pressure overload-induced heart failure. Nat. Commun. 8, 14680. 10.1038/ncomms14680 28262700 PMC5343521

[B92] Karger A. B. Nomura S. O. Guan W. Garg P. K. Tison G. H. Szklo M. (2025). Association between elevated total homocysteine and heart failure risk in the multi‐ethnic study of atherosclerosis cohort. J. Am. Heart Assoc. 14 (5), e038168. 10.1161/JAHA.124.038168 39968806 PMC12132734

[B93] Kassiri Z. Zhong J. Guo D. Basu R. Wang X. Liu P. P. (2009). Loss of angiotensin-converting enzyme 2 accelerates maladaptive left ventricular remodeling in response to myocardial infarction. Circ. Heart Fail 2 (5), 446–455. 10.1161/CIRCHEARTFAILURE.108.840124 19808375

[B94] Keever K. R. Yakubenko V. P. Hoover D. B. (2023). Neuroimmune nexus in the pathophysiology and therapy of inflammatory disorders: role of alpha7 nicotinic acetylcholine receptors. Pharmacol. Res. 191, 106758. 10.1016/j.phrs.2023.106758 37028776 PMC13134767

[B95] Keever K. R. Cui K. Casteel J. L. Singh S. Hoover D. B. Williams D. L. (2024). Cholinergic signaling *via* the alpha7 nicotinic acetylcholine receptor regulates the migration of monocyte-derived macrophages during acute inflammation. J. Neuroinflammat. 21 (1), 3. 10.1186/s12974-023-03001-7 38178134 PMC10765732

[B96] Kenny H. C. Abel E. D. (2019). Heart failure in type 2 diabetes mellitus: impact of glucose-lowering agents, heart failure therapies, and novel therapeutic strategies. Circ. Res. 124 (1), 121–141. 10.1161/CIRCRESAHA.118.311371 30605420 PMC6447311

[B97] Kessler E. L. Dal Canto E. Diez-Benavente E. van Ommen A. M. Kapteijn D. Glade M. C. (2025). Non-classical monocytes are associated with functional markers of left ventricular diastolic dysfunction and heart failure with preserved ejection fraction. Int. J. Cardiol. 429, 133161. 10.1016/j.ijcard.2025.133161 40088950

[B98] Khan M. S. Shahid I. Bennis A. Rakisheva A. Metra M. Butler J. (2024). Global epidemiology of heart failure. Nat. Rev. Cardiol. 21 (10), 717–734. 10.1038/s41569-024-01046-6 38926611

[B99] Kilianova Z. Ciznarova N. Szmicsekova K. Slobodova L. Hrabovska A. (2020). Expression of cholinesterases and their anchoring proteins in rat heart. Can. J. Physiol. Pharmacol. 98 (7), 473–476. 10.1139/cjpp-2019-0565 32017610

[B100] Kitzman D. W. Brubaker P. Morgan T. Haykowsky M. Hundley G. Kraus W. E. (2016). Effect of caloric restriction or aerobic exercise training on peak oxygen consumption and quality of life in obese older patients with heart failure with preserved ejection fraction: a randomized clinical trial. JAMA 315 (1), 36–46. 10.1001/jama.2015.17346 26746456 PMC4787295

[B101] Kjeldsen S. E. von Lueder T. G. Smiseth O. A. Wachtell K. Mistry N. Westheim A. S. (2020). Medical therapies for heart failure with preserved ejection fraction. Hypertension 75 (1), 23–32. 10.1161/HYPERTENSIONAHA.119.14057 31786973

[B102] Kneuer J. M. Muller M. Erbe S. Kokot K. E. Rosch S. Muller-Kozarez I. (2025). Circulating immune cell signature analysis in HFPEF across species. Circ. Res. 137 (5), 682–698. 10.1161/CIRCRESAHA.125.326249 40709473 PMC12352558

[B103] Kumar P. Lim A. Poh S. L. Hazirah S. N. Chua C. J. H. Sutamam N. B. (2022). Pro-inflammatory derangement of the immuno-interactome in heart failure. Front. Immunol. 13, 817514. 10.3389/fimmu.2022.817514 35371099 PMC8964981

[B104] Lala A. Tayal U. Hamo C. E. Youmans Q. Al-Khatib S. M. Bozkurt B. (2022). Sex differences in heart failure. J. Cardiac Failure 28 (3), 477–498. 10.1016/j.cardfail.2021.10.006 34774749

[B105] Lee S. J. Bai S. K. Lee K. S. Namkoong S. Na H. J. Ha K. S. (2003). Astaxanthin inhibits nitric oxide production and inflammatory gene expression by suppressing I(kappa)B kinase-dependent NF-kappaB activation. Mol. Cells 16 (1), 97–105. 10.1016/j.tcm.2024.12.011 14503852

[B106] Lee D. S. Cardone K. M. Zhang D. Y. Tsao N. L. Abramowitz S. Sharma P. (2025a). Common-variant and rare-variant genetic architecture of heart failure across the allele-frequency spectrum. Nat. Genet. 57, 1–10. 10.1038/s41588-025-02140-2 40195560 PMC12049093

[B107] Lee D.-I. Kim S. Kang D. O. (2025b). Exploring the complex interplay between alcohol consumption and cardiovascular health: mechanisms, evidence, and future directions. Trends Cardiovasc. Med. 35 (4), 243–253. 39756716 10.1016/j.tcm.2024.12.011

[B108] Letsinger A. C. Gu Z. Yakel J. L. (2022). α7 nicotinic acetylcholine receptors in the hippocampal circuit: taming complexity. Trends Neurosci. 45 (2), 145–157. 10.1016/j.tins.2021.11.006 34916082 PMC8914277

[B109] Levick S. P. Murray D. B. Janicki J. S. Brower G. L. (2010). Sympathetic nervous system modulation of inflammation and remodeling in the hypertensive heart. Hypertension 55 (2), 270–276. 10.1161/HYPERTENSIONAHA.109.142042 20048196 PMC2823485

[B110] Li X. W. Wang H. (2006). Non-neuronal nicotinic alpha 7 receptor, a new endothelial target for revascularization. Life Sci. 78 (16), 1863–1870. 10.1016/j.lfs.2005.08.031 16280133

[B111] Li Y. F. LaCroix C. Freeling J. (2009). Specific subtypes of nicotinic cholinergic receptors involved in sympathetic and parasympathetic cardiovascular responses. Neurosci. Lett. 462 (1), 20–23. 10.1016/j.neulet.2009.06.081 19573576 PMC2745958

[B112] Li N. Bian H. Zhang J. Li X. Ji X. Zhang Y. (2010). The Th17/Treg imbalance exists in patients with heart failure with normal ejection fraction and heart failure with reduced ejection fraction. Clin. Chim. Acta 411 (23-24), 1963–1968. 10.1016/j.cca.2010.08.013 20713031

[B113] Li D. J. Evans R. G. Yang Z. W. Song S. W. Wang P. Ma X. J. (2011). Dysfunction of the cholinergic anti-inflammatory pathway mediates organ damage in hypertension. Hypertension 57 (2), 298–307. 10.1161/HYPERTENSIONAHA.110.160077 21173343

[B114] Li D. J. Huang F. Ni M. Fu H. Zhang L. S. Shen F. M. (2016). alpha7 nicotinic acetylcholine receptor relieves angiotensin II-Induced senescence in vascular smooth muscle cells by raising nicotinamide adenine dinucleotide-dependent SIRT1 activity. Arterioscler. Thromb. Vasc. Biol. 36 (8), 1566–1576. 10.1161/ATVBAHA.116.307157 27339462

[B115] Li D. J. Liu J. Hua X. Fu H. Huang F. Fei Y. B. (2018). Nicotinic acetylcholine receptor alpha7 subunit improves energy homeostasis and inhibits inflammation in nonalcoholic fatty liver disease. Metabolism 79, 52–63. 10.1016/j.metabol.2017.11.002 29129819

[B116] Li Z. Hao H. Gao Y. Wang Z. Lu W. Liu J. (2019). Expression and localization analyses of the cholinergic anti-inflammatory pathway and alpha7nAchR in different tissues of rats with rheumatoid arthritis. Acta Histochem. 121 (6), 742–749. 10.1016/j.acthis.2019.07.001 31279484

[B117] Li C. Qin D. Hu J. Yang Y. Hu D. Yu B. (2022). Inflamed adipose tissue: a culprit underlying obesity and heart failure with preserved ejection fraction. Front. Immunol. 13, 947147. 10.3389/fimmu.2022.947147 36483560 PMC9723346

[B118] Liu J. J. Li D. L. Zhou J. Sun L. Zhao M. Kong S. S. (2011). Acetylcholine prevents angiotensin II-induced oxidative stress and apoptosis in H9c2 cells. Apoptosis 16 (1), 94–103. 10.1007/s10495-010-0549-x 20963497

[B119] Liu A. J. Zang P. Guo J. M. Wang W. Dong W. Z. Guo W. (2012). Involvement of acetylcholine-alpha7nAChR in the protective effects of arterial baroreflex against ischemic stroke. CNS Neurosci. Ther. 18 (11), 918–926. 10.1111/cns.12011 23106973 PMC6493387

[B120] Liu T. Zhang L. Joo D. Sun S. C. (2017a). NF-kappaB signaling in inflammation. Signal Transduct. Target Ther. 2, 17023. 10.1038/sigtrans.2017.23 29158945 PMC5661633

[B121] Liu L. Wu H. Cao Q. Guo Z. Ren A. Dai Q. (2017b). Stimulation of Alpha7 nicotinic acetylcholine receptor attenuates nicotine-induced upregulation of MMP, MCP-1, and RANTES through modulating ERK1/2/AP-1 signaling pathway in RAW264.7 and MOVAS cells. Mediat. Inflamm. 2017, 2401027. 10.1155/2017/2401027 29348704 PMC5733626

[B122] Liu E. Y. L. Xia Y. Kong X. Guo M. S. S. Yu A. X. D. Zheng B. Z. Y. (2020). Interacting with alpha 7 nAChR is a new mechanism for AChE to enhance the inflammatory response in macrophages. Acta Pharm. Sin. B 10 (10), 1926–1942. 10.1016/j.apsb.2020.05.005 33163344 PMC7606108

[B123] Liu C. M. C. Wang X. Gentile C. (2024). Protective role of acetylcholine and the cholinergic system in the injured heart. iScience 27 (9), 110726. 10.1016/j.isci.2024.110726 39280620 PMC11402255

[B124] Lu B. Kwan K. Levine Y. A. Olofsson P. S. Yang H. Li J. (2014). alpha7 nicotinic acetylcholine receptor signaling inhibits inflammasome activation by preventing mitochondrial DNA release. Mol. Med. 20 (1), 350–358. 10.2119/molmed.2013.00117 24849809 PMC4153835

[B125] Ma C. X. Ma X. N. Guan C. H. Li Y. D. Mauricio D. Fu S. B. (2022). Cardiovascular disease in type 2 diabetes mellitus: progress toward personalized management. Cardiovasc Diabetol. 21 (1), 74. 10.1186/s12933-022-01516-6 35568946 PMC9107726

[B126] Maggi F. Rosellini A. Spezia P. G. Focosi D. Macera L. Lai M. (2021). Nicotine upregulates ACE2 expression and increases competence for SARS-CoV-2 in human pneumocytes. ERJ Open Res. 7 (2), 00713-2020. 10.1183/23120541.00713-2020 33850935 PMC7942220

[B127] Manetti D. Dei S. Arias H. R. Braconi L. Gabellini A. Teodori E. (2023). Recent advances in the discovery of nicotinic acetylcholine receptor allosteric modulators. Molecules 28 (3), 1270. 10.3390/molecules28031270 36770942 PMC9920195

[B128] Mashimo M. Komori M. Matsui Y. Y. Murase M. X. Fujii T. Takeshima S. (2019). Distinct roles of alpha7 nAChRs in antigen-presenting cells and CD4(+) T cells in the regulation of T cell differentiation. Front. Immunol. 10, 1102. 10.3389/fimmu.2019.01102 31214160 PMC6554293

[B129] Mazidi M. Rezaie P. Gao H. K. Kengne A. P. (2017). Effect of sodium-glucose Cotransport-2 inhibitors on blood pressure in people with type 2 diabetes mellitus: a systematic review and meta-analysis of 43 randomized control trials with 22 528 patients. J. Am. Heart Assoc. 6 (6). 10.1161/JAHA.116.004007 28546454 PMC5669140

[B130] McDonagh T. A. Metra M. Adamo M. Gardner R. S. Baumbach A. Böhm M. (2021). 2021 ESC guidelines for the diagnosis and treatment of acute and chronic heart failure: developed by the task force for the diagnosis and treatment of acute and chronic heart failure of the European Society of Cardiology (ESC) with the special contribution of the Heart Failure Association (HFA) of the ESC. Eur. Heart J. 42 (36), 3599–3726. 10.1093/eurheartj/ehab368 34447992

[B131] Mesquita T. Lin Y. N. Ibrahim A. (2021). Chronic low-grade inflammation in heart failure with preserved ejection fraction. Aging Cell 20 (9), e13453. 10.1111/acel.13453 34382743 PMC8441359

[B132] Mishra S. Kass D. A. (2021). Cellular and molecular pathobiology of heart failure with preserved ejection fraction. Nat. Rev. Cardiol. 18 (6), 400–423. 10.1038/s41569-020-00480-6 33432192 PMC8574228

[B133] Mizrachi T. Marsha O. Brusin K. Ben-David Y. Thakur G. A. Vaknin-Dembinsky A. (2021). Suppression of neuroinflammation by an allosteric agonist and positive allosteric modulator of the alpha7 nicotinic acetylcholine receptor GAT107. J. Neuroinflammation 18 (1), 99. 10.1186/s12974-021-02149-4 33902624 PMC8077754

[B134] Mohammed S. F. Hussain S. Mirzoyev S. A. Edwards W. D. Maleszewski J. J. Redfield M. M. (2015). Coronary microvascular rarefaction and myocardial fibrosis in heart failure with preserved ejection fraction. Circulation 131 (6), 550–559. 10.1161/CIRCULATIONAHA.114.009625 25552356 PMC4324362

[B135] Mueller A. Starobova H. Inserra M. C. Jin A. H. Deuis J. R. Dutertre S. (2015). Alpha-conotoxin MrIC is a biased agonist at alpha7 nicotinic acetylcholine receptors. Biochem. Pharmacol. 94 (2), 155–163. 10.1016/j.bcp.2015.01.011 25646788

[B136] Muller D. N. Dechend R. Mervaala E. M. Park J. K. Schmidt F. Fiebeler A. (2000). NF-kappaB inhibition ameliorates angiotensin II-induced inflammatory damage in rats. Hypertension 35 (1 Pt 2), 193–201. 10.1161/01.hyp.35.1.193 10642297

[B137] Munoz-Durango N. Fuentes C. A. Castillo A. E. Gonzalez-Gomez L. M. Vecchiola A. Fardella C. E. (2016). Role of the renin-angiotensin-aldosterone system beyond blood pressure regulation: molecular and cellular mechanisms involved in end-organ damage during arterial hypertension. Int. J. Mol. Sci. 17 (7). 10.3390/ijms17070797 27347925 PMC4964362

[B138] Nadorp B. Soreq H. (2015). Gut feeling: microRNA discriminators of the intestinal TLR9-cholinergic links. Int. Immunopharmacol. 29 (1), 8–14. 10.1016/j.intimp.2015.04.058 26003847 PMC4646847

[B139] Naing P. Forrester D. Kangaharan N. Muthumala A. Mon Myint S. Playford D. (2019). Heart failure with preserved ejection fraction: a growing global epidemic. Aust. J. Gen. Pract. 48 (7), 465–471. 10.31128/AJGP-03-19-4873 31256507

[B140] Nebuwa C. N. Iyeku A. C. Ndakotsu A. K. Ugoala O. S. Mandal D. Uwumiro F. (2024). Impact of dyslipidemia on clinical outcomes among patients with heart failure: a nationwide retrospective analysis. J. Am. Coll. Cardiol. 83 (13_Suppl), 633. 10.1016/s0735-1097(24)02623-8

[B141] Nevers T. Salvador A. M. Velazquez F. Ngwenyama N. Carrillo-Salinas F. J. Aronovitz M. (2017). Th1 effector T cells selectively orchestrate cardiac fibrosis in nonischemic heart failure. J. Exp. Med. 214 (11), 3311–3329. 10.1084/jem.20161791 28970239 PMC5679176

[B142] Nizri E. Hamra-Amitay Y. Sicsic C. Lavon I. Brenner T. (2006). Anti-inflammatory properties of cholinergic up-regulation: a new role for acetylcholinesterase inhibitors. Neuropharmacology 50 (5), 540–547. 10.1016/j.neuropharm.2005.10.013 16336980

[B143] Nizri E. Irony-Tur-Sinai M. Faranesh N. Lavon I. Lavi E. Weinstock M. (2008). Suppression of neuroinflammation and immunomodulation by the acetylcholinesterase inhibitor rivastigmine. J. Neuroimmunol. 203 (1), 12–22. 10.1016/j.jneuroim.2008.06.018 18692909

[B144] Nizri E. Irony-Tur-Sinai M. Lory O. Orr-Urtreger A. Lavi E. Brenner T. (2009). Activation of the cholinergic anti-inflammatory system by nicotine attenuates neuroinflammation *via* suppression of Th1 and Th17 responses. J. Immunol. 183 (10), 6681–6688. 10.4049/jimmunol.0902212 19846875

[B145] Norhammar A. Bodegard J. Vanderheyden M. Tangri N. Karasik A. Maggioni A. P. (2023). Prevalence, outcomes and costs of a contemporary, multinational population with heart failure. Heart 109 (7), 548–556. 10.1136/heartjnl-2022-321702 36781285 PMC10086499

[B146] Noviello C. M. Gharpure A. Mukhtasimova N. Cabuco R. Baxter L. Borek D. (2021). Structure and gating mechanism of the α7 nicotinic acetylcholine receptor. Cell 184 (8), 2121–2134. e13. 10.1016/j.cell.2021.02.049 33735609 PMC8135066

[B147] Odajima S. Tanaka H. Fujimoto W. Kuroda K. Yamashita S. Imanishi J. (2022). Efficacy of renin-angiotensin-aldosterone-system inhibitors for heart failure with preserved ejection fraction and left ventricular hypertrophy-from the KUNIUMI Registry Acute Cohort. J. Cardiol. 79 (6), 703–710. 10.1016/j.jjcc.2021.12.002 34924235

[B148] Oeckinghaus A. Hayden M. S. Ghosh S. (2011). Crosstalk in NF-kappaB signaling pathways. Nat. Immunol. 12 (8), 695–708. 10.1038/ni.2065 21772278

[B149] Panagis G. Kastellakis A. Spyraki C. Nomikos G. (2000). Effects of methyllycaconitine (MLA), an alpha 7 nicotinic receptor antagonist, on nicotine- and cocaine-induced potentiation of brain stimulation reward. Psychopharmacol. Berl. 149 (4), 388–396. 10.1007/s002130000384 10867966

[B150] Pandey A. LaMonte M. Klein L. Ayers C. Psaty B. M. Eaton C. B. (2017). Relationship between physical activity, body mass index, and risk of heart failure. J. Am. Coll. Cardiol. 69 (9), 1129–1142. 10.1016/j.jacc.2016.11.081 28254175 PMC5848099

[B151] Papke R. L. Horenstein N. A. (2021). Therapeutic targeting of alpha7 nicotinic acetylcholine receptors. Pharmacol. Rev. 73 (3), 1118–1149. 10.1124/pharmrev.120.000097 34301823 PMC8318519

[B152] Papke R. L. Horenstein N. A. Kulkarni A. R. Stokes C. Corrie L. W. Maeng C. Y. (2014). The activity of GAT107, an allosteric activator and positive modulator of alpha7 nicotinic acetylcholine receptors (nAChR), is regulated by aromatic amino acids that span the subunit interface. J. Biol. Chem. 289 (7), 4515–4531. 10.1074/jbc.M113.524603 24362025 PMC3924313

[B153] Patel V. B. Clarke N. Wang Z. Fan D. Parajuli N. Basu R. (2014). Angiotensin II induced proteolytic cleavage of myocardial ACE2 is mediated by TACE/ADAM-17: a positive feedback mechanism in the RAS. J. Mol. Cell Cardiol. 66, 167–176. 10.1016/j.yjmcc.2013.11.017 24332999

[B154] Patel V. B. Zhong J. C. Grant M. B. Oudit G. Y. (2016). Role of the ACE2/Angiotensin 1-7 axis of the renin-angiotensin system in heart failure. Circ. Res. 118 (8), 1313–1326. 10.1161/CIRCRESAHA.116.307708 27081112 PMC4939482

[B155] Paulus W. J. Tschope C. (2013). A novel paradigm for heart failure with preserved ejection fraction: comorbidities drive myocardial dysfunction and remodeling through coronary microvascular endothelial inflammation. J. Am. Coll. Cardiol. 62 (4), 263–271. 10.1016/j.jacc.2013.02.092 23684677

[B156] Pavlov V. A. Tracey K. J. (2012). The vagus nerve and the inflammatory reflex--linking immunity and metabolism. Nat. Rev. Endocrinol. 8 (12), 743–754. 10.1038/nrendo.2012.189 23169440 PMC4082307

[B157] Peh Z. H. Dihoum A. Hutton D. Arthur J. S. C. Rena G. Khan F. (2023). Inflammation as a therapeutic target in heart failure with preserved ejection fraction. Front. Cardiovasc Med. 10, 1125687. 10.3389/fcvm.2023.1125687 37456816 PMC10339321

[B158] Petrie J. R. Guzik T. J. Touyz R. M. (2018). Diabetes, hypertension, and cardiovascular disease: clinical insights and vascular mechanisms. Can. J. Cardiol. 34 (5), 575–584. 10.1016/j.cjca.2017.12.005 29459239 PMC5953551

[B159] Petrov K. A. Girard E. Nikitashina A. D. Colasante C. Bernard V. Nurullin L. (2014). Schwann cells sense and control acetylcholine spillover at the neuromuscular junction by alpha7 nicotinic receptors and butyrylcholinesterase. J. Neurosci. 34 (36), 11870–11883. 10.1523/JNEUROSCI.0329-14.2014 25186736 PMC6608466

[B160] Petrov K. Lenina O. Leroy J. Bernard V. Germain T. Truong C. (2025). An alpha7 nicotinic and GABA(B) receptor-mediated pathway controls acetylcholine release in the tripartite neuromuscular junction. J. Physiol. 603 (2), 507–527. 10.1113/JP287243 39740234 PMC11737540

[B161] Pitt B. Pfeffer M. A. Assmann S. F. Boineau R. Anand I. S. Claggett B. (2014). Spironolactone for heart failure with preserved ejection fraction. N. Engl. J. Med. 370 (15), 1383–1392. 10.1056/NEJMoa1313731 24716680

[B162] Prausmuller S. Weidenhammer A. Heitzinger G. Spinka G. Goliasch G. Arfsten H. (2023). Obesity in heart failure with preserved ejection fraction with and without diabetes: risk factor or innocent bystander? Eur. J. Prev. Cardiol. 30 (12), 1247–1254. 10.1093/eurjpc/zwad140 37210596

[B163] Prickaerts J. van Goethem N. P. Chesworth R. Shapiro G. Boess F. G. Methfessel C. (2012). EVP-6124, a novel and selective alpha7 nicotinic acetylcholine receptor partial agonist, improves memory performance by potentiating the acetylcholine response of alpha7 nicotinic acetylcholine receptors. Neuropharmacology 62 (2), 1099–1110. 10.1016/j.neuropharm.2011.10.024 22085888

[B164] Qasem A. M. Rowan M. G. Sanders V. R. Millar N. S. Blagbrough I. S. (2023). Synthesis and antagonist activity of methyllycaconitine analogues on human α7 nicotinic acetylcholine receptors. ACS Bio Med Chem Au 3 (2), 147–157. 10.1021/acsbiomedchemau.2c00057 37096031 PMC10119942

[B165] Rasooly D. Moonesinghe R. Littrell K. Hull L. Khoury M. J. (2023). Association between a first‐degree family history and self‐reported personal history of obesity, diabetes, and heart and blood conditions: results from the all of Us Research Program. J. Am. Heart Assoc. 12 (22), e030779. 10.1161/JAHA.123.030779 37947093 PMC10727309

[B166] Rathouz M. M. Berg D. K. (1994). Synaptic-type acetylcholine receptors raise intracellular calcium levels in neurons by two mechanisms. J. Neurosci. 14 (11 Pt 2), 6935–6945. 10.1523/JNEUROSCI.14-11-06935.1994 7965089 PMC6577244

[B167] Rayner J. J. Abdesselam I. Pan J. Lewis A. J. Rider O. J. (2025). Obesity and heart failure: exploring the cardiometabolic axis. Cardiovasc. Res., cvaf090. 121 (8), 1173–1186. 10.1093/cvr/cvaf090 40458047 PMC12310286

[B168] Reardon C. Duncan G. S. Brustle A. Brenner D. Tusche M. W. Olofsson P. S. (2013). Lymphocyte-derived ACh regulates local innate but not adaptive immunity. Proc. Natl. Acad. Sci. U. S. A. 110 (4), 1410–1415. 10.1073/pnas.1221655110 23297238 PMC3557089

[B169] Reichrath S. Reichrath J. Moussa A. T. Meier C. Tschernig T. (2016). Targeting the non-neuronal cholinergic system in macrophages for the management of infectious diseases and cancer: challenge and promise. Cell Death Discov. 2, 16063. 10.1038/cddiscovery.2016.63 27785369 PMC5066265

[B170] Rist A. Sevre K. Wachtell K. Devereux R. B. Aurigemma G. P. Smiseth O. A. (2024). The current best drug treatment for hypertensive heart failure with preserved ejection fraction. Eur. J. Inter. Med. 120, 3–10. 10.1016/j.ejim.2023.10.008 37865559

[B171] Roche C. Besnier M. Cassel R. Harouki N. Coquerel D. Guerrot D. (2015). Soluble epoxide hydrolase inhibition improves coronary endothelial function and prevents the development of cardiac alterations in obese insulin-resistant mice. Am. J. Physiol. Heart Circ. Physiol. 308 (9), H1020–H1029. 10.1152/ajpheart.00465.2014 25724490 PMC4551118

[B172] Rosas-Ballina M. Olofsson P. S. Ochani M. Valdes-Ferrer S. I. Levine Y. A. Reardon C. (2011). Acetylcholine-synthesizing T cells relay neural signals in a vagus nerve circuit. Science 334 (6052), 98–101. 10.1126/science.1209985 21921156 PMC4548937

[B173] Roth G. A. Mensah G. A. Johnson C. O. Addolorato G. Ammirati E. Baddour L. M. (2020). Global burden of cardiovascular diseases and risk factors, 1990–2019: update from the GBD 2019 study. J. Am. Coll. Cardiol. 76 (25), 2982–3021. 10.1016/j.jacc.2020.11.010 33309175 PMC7755038

[B174] Ruan Y. Yu Y. Wu M. Jiang Y. Qiu Y. Ruan S. (2024). The renin-angiotensin-aldosterone system: an old tree sprouts new shoots. Cell Signal 124, 111426. 10.1016/j.cellsig.2024.111426 39306263

[B175] Saeed R. W. Varma S. Peng-Nemeroff T. Sherry B. Balakhaneh D. Huston J. (2005). Cholinergic stimulation blocks endothelial cell activation and leukocyte recruitment during inflammation. J. Exp. Med. 201 (7), 1113–1123. 10.1084/jem.20040463 15809354 PMC2213139

[B176] Samsky M. D. Hellkamp A. Hiatt W. R. Fowkes F. G. R. Baumgartner I. Berger J. S. (2021). Association of heart failure with outcomes among patients with peripheral artery disease: insights from EUCLID. J. Am. Heart Assoc. 10 (12), e018684. 10.1161/JAHA.120.018684 34056910 PMC8477881

[B177] Sanders-van Wijk S. Tromp J. Beussink-Nelson L. Hage C. Svedlund S. Saraste A. (2020). Proteomic evaluation of the comorbidity-inflammation paradigm in heart failure with preserved ejection fraction: results from the PROMIS-HFPEF study. Circulation 142 (21), 2029–2044. 10.1161/CIRCULATIONAHA.120.045810 33034202 PMC7686082

[B178] Sato K. Z. Fujii T. Watanabe Y. Yamada S. Ando T. Kazuko F. (1999). Diversity of mRNA expression for muscarinic acetylcholine receptor subtypes and neuronal nicotinic acetylcholine receptor subunits in human mononuclear leukocytes and leukemic cell lines. Neurosci. Lett. 266 (1), 17–20. 10.1016/s0304-3940(99)00259-1 10336173

[B179] Savarese G. Becher P. M. Lund L. H. Seferovic P. Rosano G. M. Coats A. J. (2022). Global burden of heart failure: a comprehensive and updated review of epidemiology. Cardiovasc. Res. 118 (17), 3272–3287. 10.1093/cvr/cvac013 35150240

[B180] Savitz S. T. Leong T. Sung S. H. Lee K. Rana J. S. Tabada G. (2021). Contemporary reevaluation of race and ethnicity with outcomes in heart failure. J. Am. Heart Assoc. 10 (3), e016601. 10.1161/JAHA.120.016601 33474975 PMC7955425

[B181] Savji N. Meijers W. C. Bartz T. M. Bhambhani V. Cushman M. Nayor M. (2018). The Association of obesity and cardiometabolic traits with incident HFPEF and HFREF. JACC Heart Fail 6 (8), 701–709. 10.1016/j.jchf.2018.05.018 30007554 PMC6076337

[B182] Saw E. L. Pearson J. T. Schwenke D. O. Munasinghe P. E. Tsuchimochi H. Rawal S. (2021). Activation of the cardiac non-neuronal cholinergic system prevents the development of diabetes-associated cardiovascular complications. Cardiovasc Diabetol. 20 (1), 50. 10.1186/s12933-021-01231-8 33618724 PMC7898760

[B183] Schiattarella G. G. Rodolico D. Hill J. A. (2021). Metabolic inflammation in heart failure with preserved ejection fraction. Cardiovasc Res. 117 (2), 423–434. 10.1093/cvr/cvaa217 32666082 PMC8599724

[B184] Schloss M. J. Hulsmans M. Rohde D. Lee I. H. Severe N. Foy B. H. (2022). B lymphocyte-derived acetylcholine limits steady-state and emergency hematopoiesis. Nat. Immunol. 23 (4), 605–618. 10.1038/s41590-022-01165-7 35352063 PMC8989652

[B185] Seravalle G. Quarti-Trevano F. Dell'Oro R. Gronda E. Spaziani D. Facchetti R. (2019). Sympathetic and baroreflex alterations in congestive heart failure with preserved, midrange and reduced ejection fraction. J. Hypertens. 37 (2), 443–448. 10.1097/HJH.0000000000001856 30020242

[B186] Shaw S. Bencherif M. Marrero M. B. (2003). Angiotensin II blocks nicotine-mediated neuroprotection against beta-amyloid (1-42) *via* activation of the tyrosine phosphatase SHP-1. J. Neurosci. 23 (35), 11224–11228. 10.1523/JNEUROSCI.23-35-11224.2003 14657181 PMC6741058

[B187] Simon T. Kirk J. Dolezalova N. Guyot M. Panzolini C. Bondue A. (2023). The cholinergic anti-inflammatory pathway inhibits inflammation without lymphocyte relay. Front. Neurosci. 17, 1125492. 10.3389/fnins.2023.1125492 37123375 PMC10140439

[B188] Singhania N. Bansal S. Mohandas S. Nimmatoori D. P. Ejaz A. A. Singhania G. (2020). Role of renin-angiotensin-aldosterone system inhibitors in heart failure and chronic kidney disease. Drugs Context 9. 10.7573/dic.2020-7-3 33240389 PMC7673621

[B189] Sitapara R. A. Gauthier A. G. Valdes-Ferrer S. I. Lin M. Patel V. Wang M. (2020). The alpha7 nicotinic acetylcholine receptor agonist, GTS-21, attenuates hyperoxia-induced acute inflammatory lung injury by alleviating the accumulation of HMGB1 in the airways and the circulation. Mol. Med. 26 (1), 63. 10.1186/s10020-020-00177-z 32600307 PMC7322715

[B190] Solomon S. D. McMurray J. J. V. Anand I. S. Ge J. Lam C. S. P. Maggioni A. P. (2019). Angiotensin-neprilysin inhibition in heart failure with preserved ejection fraction. N. Engl. J. Med. 381 (17), 1609–1620. 10.1056/NEJMoa1908655 31475794

[B191] Solomon S. D. de Boer R. A. DeMets D. Hernandez A. F. Inzucchi S. E. Kosiborod M. N. (2021). Dapagliflozin in heart failure with preserved and mildly reduced ejection fraction: rationale and design of the DELIVER trial. Eur. J. Heart Fail 23 (7), 1217–1225. 10.1002/ejhf.2249 34051124 PMC8361994

[B192] Souza A. C. P. Souza C. M. Amaral C. L. Lemes S. F. Santucci L. F. Milanski M. (2019). Short-term high-fat diet consumption reduces hypothalamic expression of the nicotinic acetylcholine receptor alpha7 subunit (alpha7nAChR) and affects the anti-inflammatory response in a mouse model of sepsis. Front. Immunol. 10, 565. 10.3389/fimmu.2019.00565 30967878 PMC6438922

[B193] Strassheim D. Dempsey E. C. Gerasimovskaya E. Stenmark K. Karoor V. (2019). Role of inflammatory cell subtypes in heart failure. J. Immunol. Res. 2019, 2164017. 10.1155/2019/2164017 31565659 PMC6745095

[B194] Styron J. F. Jois-Bilowich P. Starling R. Hobbs R. E. Kontos M. C. Pang P. S. (2009). Initial emergency department systolic blood pressure predicts left ventricular systolic function in acute decompensated heart failure. Congest. Heart Fail 15 (1), 9–13. 10.1111/j.1751-7133.2008.00047.x 19187401

[B195] Su Y. Zhang W. Zhang R. Yuan Q. Wu R. Liu X. (2022). Activation of cholinergic anti-inflammatory pathway ameliorates cerebral and cardiac dysfunction after intracerebral hemorrhage through autophagy. Front. Immunol. 13, 870174. 10.3389/fimmu.2022.870174 35812436 PMC9260497

[B196] Sun Y. Jia D. Xue M. Huang Z. Huang C. (2022). Trifluoro-icaritin alleviates chronic inflammatory pain through alpha7nAChR-mediated suppression of HMGB1/NF-kappaB signaling in the spinal cord of rats. Brain Res. Bull. 183, 13–26. 10.1016/j.brainresbull.2022.02.014 35202753

[B197] Tam M. C. Lee R. Cascino T. M. Konerman M. C. Hummel S. L. (2017). Current perspectives on systemic hypertension in heart failure with preserved ejection fraction. Curr. Hypertens. Rep. 19 (2), 12. 10.1007/s11906-017-0709-2 28233237 PMC5503692

[B198] Targosova K. Kucera M. Fazekas T. Kilianova Z. Stankovicova T. Hrabovska A. (2024). alpha7 nicotinic receptors play a role in regulation of cardiac hemodynamics. J. Neurochem. 168 (4), 414–427. 10.1111/jnc.15821 37017608

[B199] Thibodeau J. T. Drazner M. H. (2018). The role of the clinical examination in patients with heart failure. JACC Heart Fail. 6 (7), 543–551. 10.1016/j.jchf.2018.04.005 29885957

[B200] Tromp J. Paniagua S. M. Lau E. S. Allen N. B. Blaha M. J. Gansevoort R. T. (2021). Age dependent associations of risk factors with heart failure: pooled population based cohort study. Bmj 372, n461. 10.1136/bmj.n461 33758001 PMC7986583

[B201] Truong L. D. Trostel J. Garcia G. E. (2015). Absence of nicotinic acetylcholine receptor alpha7 subunit amplifies inflammation and accelerates onset of fibrosis: an inflammatory kidney model. FASEB J. 29 (8), 3558–3570. 10.1096/fj.14-262493 25985801 PMC4511204

[B202] Ulleryd M. A. Mjörnstedt F. Panagaki D. Yang L. J. Engevall K. Gutiérrez S. (2019). Stimulation of alpha 7 nicotinic acetylcholine receptor (α7nAChR) inhibits atherosclerosis *via* immunomodulatory effects on myeloid cells. Atherosclerosis 287, 122–133. 10.1016/j.atherosclerosis.2019.06.903 31260875

[B203] van Dalen B. M. Chin J. F. Motiram P. A. Hendrix A. Emans M. E. Brugts J. J. (2025). Challenges in the diagnosis of heart failure with preserved ejection fraction in individuals with obesity. Cardiovasc Diabetol. 24 (1), 71. 10.1186/s12933-025-02612-z 39920805 PMC11806779

[B204] Vang A. da Silva Goncalves Bos D. Fernandez-Nicolas A. Zhang P. Morrison A. R. Mancini T. J. (2021). alpha7 nicotinic acetylcholine receptor mediates right ventricular fibrosis and diastolic dysfunction in pulmonary hypertension. JCI Insight 6 (12), e142945. 10.1172/jci.insight.142945 33974567 PMC8262476

[B205] Vasan R. S. Xanthakis V. Lyass A. Andersson C. Tsao C. Cheng S. (2018). Epidemiology of left ventricular systolic dysfunction and heart failure in the framingham study: an echocardiographic study over 3 decades. JACC Cardiovasc Imaging 11 (1), 1–11. 10.1016/j.jcmg.2017.08.007 28917679 PMC5756128

[B206] Vieira-Alves I. Coimbra-Campos L. M. Sancho M. Da Silva R. F. Cortes S. F. Lemos V. S. (2020). Role of the α7 nicotinic acetylcholine receptor in the pathophysiology of atherosclerosis. Front. Physiol. 11, 621769. 10.3389/fphys.2020.621769 33424644 PMC7785985

[B207] Villeda-Gonzalez J. D. Gomez-Olivares J. L. Baiza-Gutman L. A. (2024). New paradigms in the study of the cholinergic system and metabolic diseases: Acetyl-and-butyrylcholinesterase. J. Cell Physiol. 239 (8), e31274. 10.1002/jcp.31274 38605655

[B208] Wada T. Naito M. Kenmochi H. Tsuneki H. Sasaoka T. (2007). Chronic nicotine exposure enhances insulin-induced mitogenic signaling *via* up-regulation of alpha7 nicotinic receptors in isolated rat aortic smooth muscle cells. Endocrinology 148 (2), 790–799. 10.1210/en.2006-0907 17068140

[B209] Wahid A. Chen W. Wang X. Tang X. (2021). High-mobility group box 1 serves as an inflammation driver of cardiovascular disease. Biomed. Pharmacother. 139, 111555. 10.1016/j.biopha.2021.111555 33865014

[B210] Wang H. Yu M. Ochani M. Amella C. A. Tanovic M. Susarla S. (2003). Nicotinic acetylcholine receptor alpha7 subunit is an essential regulator of inflammation. Nature 421 (6921), 384–388. 10.1038/nature01339 12508119

[B211] Watson M. Dardari Z. Kianoush S. Hall M. E. DeFilippis A. P. Keith R. J. (2019). Relation between cigarette smoking and heart failure (from the multiethnic study of atherosclerosis). Am. J. Cardiol. 123 (12), 1972–1977. 10.1016/j.amjcard.2019.03.015 30967285 PMC6529241

[B212] Wessler I. Kirkpatrick C. J. (2008). Acetylcholine beyond neurons: the non-neuronal cholinergic system in humans. Br. J. Pharmacol. 154 (8), 1558–1571. 10.1038/bjp.2008.185 18500366 PMC2518461

[B213] Westermann D. Lindner D. Kasner M. Zietsch C. Savvatis K. Escher F. (2011). Cardiac inflammation contributes to changes in the extracellular matrix in patients with heart failure and normal ejection fraction. Circ. Heart Fail 4 (1), 44–52. 10.1161/CIRCHEARTFAILURE.109.931451 21075869

[B214] Wishka D. G. Walker D. P. Yates K. M. Reitz S. C. Jia S. Myers J. K. (2006). Discovery of N-[(3 R)-1-Azabicyclo [2.2. 2] oct-3-yl] furo [2, 3-c] pyridine-5-carboxamide, an agonist of the α7 nicotinic acetylcholine receptor, for the potential treatment of cognitive deficits in schizophrenia: synthesis and structure− activity relationship. J. Med. Chem. 49 (14), 4425–4436. 10.1021/jm0602413 16821801

[B215] Wittenberg R. E. Wolfman S. L. De Biasi M. Dani J. A. (2020). Nicotinic acetylcholine receptors and nicotine addiction: a brief introduction. Neuropharmacology 177, 108256. 10.1016/j.neuropharm.2020.108256 32738308 PMC7554201

[B216] Wu S. Zhao H. Luo H. Xiao X. Zhang H. Li T. (2014). GTS-21, an alpha7-nicotinic acetylcholine receptor agonist, modulates Th1 differentiation in CD4(+) T cells from patients with rheumatoid arthritis. Exp. Ther. Med. 8 (2), 557–562. 10.3892/etm.2014.1754 25009619 PMC4079428

[B217] Wu Y. J. Wang L. Ji C. F. Gu S. F. Yin Q. Zuo J. (2021a). The role of alpha7nAChR-Mediated cholinergic anti-inflammatory pathway in immune cells. Inflammation 44 (3), 821–834. 10.1007/s10753-020-01396-6 33405021

[B218] Wu S. J. Shi Z. W. Wang X. Ren F. F. Xie Z. Y. Lei L. (2021b). Activation of the cholinergic anti-inflammatory pathway attenuated angiotension II-Dependent hypertension and renal injury. Front. Pharmacol. 12, 593682. 10.3389/fphar.2021.593682 33815099 PMC8010129

[B219] Wu K. Cheng T. Zhai Z. Jiang C. Zhou X. (2023). Alpha 7-nicotinic cholinoceptor regulation of pericyte-containing retinal capillaries. Br. J. Pharmacol. 180 (17), 2196–2213. 10.1111/bph.16067 36908011

[B220] Wu S. Xie Y. Jiang Y. Zhang X. Zhou Y. Zuo X. (2025). GTS-21 modulates rheumatoid arthritis Th17 and Th2 lymphocyte subset differentiation through the ɑ7nAch receptor. Clin. Rheumatol. 44, 1–10. 10.1007/s10067-025-07320-3 39812970

[B221] Xia N. Strand S. Schlufter F. Siuda D. Reifenberg G. Kleinert H. (2013). Role of SIRT1 and FOXO factors in eNOS transcriptional activation by resveratrol. Nitric Oxide 32, 29–35. 10.1016/j.niox.2013.04.001 23583951

[B222] Xie H. Yepuri N. Meng Q. Dhawan R. Leech C. A. Chepurny O. G. (2020). Therapeutic potential of alpha7 nicotinic acetylcholine receptor agonists to combat obesity, diabetes, and inflammation. Rev. Endocr. Metab. Disord. 21 (4), 431–447. 10.1007/s11154-020-09584-3 32851581 PMC7572644

[B223] Xu M. Xue R.-Q. Lu Y. Yong S.-Y. Wu Q. Cui Y.-L. (2018). Choline ameliorates cardiac hypertrophy by regulating metabolic remodelling and UPRmt through SIRT3-AMPK pathway. Cardiovasc. Res. 115 (3), 530–545. 10.1093/cvr/cvy217 30165480

[B224] Xu Z. Q. Zhang W. J. Su D. F. Zhang G. Q. Miao C. Y. (2021). Cellular responses and functions of alpha7 nicotinic acetylcholine receptor activation in the brain: a narrative review. Ann. Transl. Med. 9 (6), 509. 10.21037/atm-21-273 33850906 PMC8039675

[B225] Yamamoto K. Origasa H. Hori M. Investigators J. D. (2013). Effects of carvedilol on heart failure with preserved ejection fraction: the Japanese Diastolic Heart Failure Study (J-DHF). Eur. J. Heart Fail 15 (1), 110–118. 10.1093/eurjhf/hfs141 22983988

[B226] Yang T. Xiao T. Sun Q. Wang K. (2017). The current agonists and positive allosteric modulators of α7 nAChR for CNS indications in clinical trials. Acta Pharm. Sin. B 7 (6), 611–622. 10.1016/j.apsb.2017.09.001 29159020 PMC5687317

[B227] Yang H. Liu H. Zeng Q. Imperato G. H. Addorisio M. E. Li J. (2019). Inhibition of HMGB1/RAGE-mediated endocytosis by HMGB1 antagonist box A, anti-HMGB1 antibodies, and cholinergic agonists suppresses inflammation. Mol. Med. 25 (1), 13. 10.1186/s10020-019-0081-6 30975096 PMC6460792

[B228] Yang H. J. Kong B. Shuai W. Zhang J. J. Huang H. (2020a). Knockout of MD1 contributes to sympathetic hyperactivity and exacerbates ventricular arrhythmias following heart failure with preserved ejection fraction *via* NLRP3 inflammasome activation. Exp. Physiol. 105 (6), 966–978. 10.1113/EP088390 32240565

[B229] Yang H. Wang H. Andersson U. (2020b). Targeting inflammation driven by HMGB1. Front. Immunol. 11, 484. 10.3389/fimmu.2020.00484 32265930 PMC7099994

[B230] Yang H. Zhu J. Fu H. Shuai W. (2024). Dapansutrile ameliorates atrial inflammation and vulnerability to atrial fibrillation in HFpEF rats. Heart Lung Circ. 33 (1), 65–77. 10.1016/j.hlc.2023.09.017 38040503

[B231] Yao C. Veleva T. Scott L. Jr. Cao S. Li L. Chen G. (2018). Enhanced cardiomyocyte NLRP3 inflammasome signaling promotes atrial fibrillation. Circulation 138 (20), 2227–2242. 10.1161/CIRCULATIONAHA.118.035202 29802206 PMC6252285

[B232] Yaxley J. P. Thambar S. V. (2015). Resistant hypertension: an approach to management in primary care. J. Fam. Med. Prim. Care 4 (2), 193–199. 10.4103/2249-4863.154630 25949966 PMC4408700

[B233] Yazawa H. Miyachi M. Furukawa M. Takahashi K. Takatsu M. Tsuboi K. (2011). Angiotensin-converting enzyme inhibition promotes coronary angiogenesis in the failing heart of Dahl salt-sensitive hypertensive rats. J. Card. Fail 17 (12), 1041–1050. 10.1016/j.cardfail.2011.09.002 22123369

[B234] Yoshikawa H. Kurokawa M. Ozaki N. Nara K. Atou K. Takada E. (2006). Nicotine inhibits the production of proinflammatory mediators in human monocytes by suppression of I-kappaB phosphorylation and nuclear factor-kappaB transcriptional activity through nicotinic acetylcholine receptor alpha7. Clin. Exp. Immunol. 146 (1), 116–123. 10.1111/j.1365-2249.2006.03169.x 16968406 PMC1809735

[B235] Zdanowski R. Krzyzowska M. Ujazdowska D. Lewicka A. Lewicki S. (2015). Role of alpha7 nicotinic receptor in the immune system and intracellular signaling pathways. Cent. Eur. J. Immunol. 40 (3), 373–379. 10.5114/ceji.2015.54602 26648784 PMC4655390

[B236] Zhang W. Sun Q. Gao X. Jiang Y. Li R. Ye J. (2013). Anti-inflammation of spirocyclopiperazinium salt compound LXM-10 targeting alpha7 nAChR and M4 mAChR and inhibiting JAK2/STAT3 pathway in rats. PLoS One 8 (6), e66895. 10.1371/journal.pone.0066895 23840548 PMC3695990

[B237] Zhang L. Zhang B. Yu Y. Wang J. Wu J. Su Y. (2021). Angiotensin II increases HMGB1 expression in the myocardium through AT1 and AT2 receptors when under pressure overload. Int. Heart J. 62 (1), 162–170. 10.1536/ihj.20-384 33455985

[B238] Zhang W. Lin H. Zou M. Yuan Q. Huang Z. Pan X. (2022a). Nicotine in inflammatory diseases: Anti-inflammatory and pro-inflammatory effects. Front. Immunol. 13, 826889. 10.3389/fimmu.2022.826889 35251010 PMC8895249

[B239] Zhang X. L. Wang T. Y. Chen Z. Wang H. W. Yin Y. Wang L. (2022b). HMGB1-Promoted neutrophil extracellular traps contribute to cardiac diastolic dysfunction in mice. J. Am. Heart Assoc. 11 (4), e023800. 10.1161/JAHA.121.023800 35156391 PMC9245819

[B240] Zhao Y. X. Yin H. Q. Yu Q. T. Qiao Y. Dai H. Y. Zhang M. X. (2010). ACE2 overexpression ameliorates left ventricular remodeling and dysfunction in a rat model of myocardial infarction. Hum. Gene Ther. 21 (11), 1545–1554. 10.1089/hum.2009.160 20507236

[B241] Zhong J. Basu R. Guo D. Chow F. L. Byrns S. Schuster M. (2010). Angiotensin-converting enzyme 2 suppresses pathological hypertrophy, myocardial fibrosis, and cardiac dysfunction. Circulation 122 (7), 717–728. 10.1161/CIRCULATIONAHA.110.955369 20679547

[B242] Zhou Z. Liu Q. Zheng M. Zuo Z. Zhang G. Shi R. (2024). Comparative study on the predictive value of TG/HDL-C, TyG and TyG-BMI indices for 5-year mortality in critically ill patients with chronic heart failure: a retrospective study. Cardiovasc. Diabetol. 23 (1), 213. 10.1186/s12933-024-02308-w 38902757 PMC11191322

[B243] Zhu W. Wang F. Hu C. Zhao Q. Zhang D. Wang X. (2024). GTS-21 attenuates ACE/ACE2 ratio and glycocalyx shedding in lipopolysaccharide-induced acute lung injury by targeting macrophage polarization derived ADAM-17. Int. Immunopharmacol. 129, 111603. 10.1016/j.intimp.2024.111603 38310766

[B244] Zisman L. S. (2005). ACE and ACE2: a tale of two enzymes. Eur. Heart J. 26 (4), 322–324. 10.1093/eurheartj/ehi043 15671047 PMC7108503

[B245] Zuo G. F. Wang L. G. Huang L. Ren Y. F. Ge Z. Hu Z. Y. (2024). TAX1BP1 downregulation by STAT3 in cardiac fibroblasts contributes to diabetes-induced heart failure with preserved ejection fraction. Biochim. Biophys. Acta Mol. Basis Dis. 1870 (2), 166979. 10.1016/j.bbadis.2023.166979 38065272

