# Correction: Alpha-7 nicotinic acetylcholine receptor: targeting the interplay between inflammation, renin-angiotensin aldosterone system, and nervous system for the novel treatment of heart failure

**DOI:** 10.3389/fphar.2026.1841172

**Published:** 2026-04-13

**Authors:** Jordan Swiderski, Laura Kate Gadanec, Stephen Hearth, Benjamin Darcy Rowlands, Andrew James Murphy, Vasso Apostolopoulos, Anthony Zulli

**Affiliations:** 1 Institute for Health and Sport (IHES), Victoria University, Melbourne, VIC, Australia; 2 School of Health Sciences, The University of Notre Dame Australia, Sydney, NSW, Australia; 3 Division of Immunometabolism, Baker Heart and Diabetes institute, Melbourne, VIC, Australia; 4 School of Health and Biomedical Science, RMIT University, Melbourne, VIC, Australia

**Keywords:** cardiovascular disease, cholinergic anti-inflammatory pathway, heart failure, hypertension, renin-angiotensin-aldosterone system, α7 nicotinic acetylcholine receptor

The **title** of this article was erroneously given as: sAlpha-7 nicotinic acetylcholine receptor: targeting the interplay between inflammation, renin-angiotensin aldosterone system, and nervous system for the novel treatment of heart failure.

The correct **title** of the article is: Alpha-7 nicotinic acetylcholine receptor: targeting the interplay between inflammation, renin-angiotensin aldosterone system, and nervous system for the novel treatment of heart failure.

The original article has been updated.

